# Integrative analysis of microRNAs and mRNAs revealed regulation of composition and metabolism in Nelore cattle

**DOI:** 10.1186/s12864-018-4514-3

**Published:** 2018-02-07

**Authors:** Gabriella B. Oliveira, Luciana C. A. Regitano, Aline S. M. Cesar, James M. Reecy, Karina Y. Degaki, Mirele D. Poleti, Andrezza M. Felício, James E. Koltes, Luiz L. Coutinho

**Affiliations:** 1 Department of Animal Science, University of São Paulo, Piracicaba, SP 13418-900 Brazil; 2 Embrapa Southeast Livestock, São Carlos, SP 13560-970 Brazil; 3 Department of Animal Science, Iowa State University, Ames, IA 50011 USA; 4 Department of Animal Science, University of Arkansas, Fayetteville, AR 72701 USA

**Keywords:** Lipids, RNA-Seq, *Bos indicus*, microRNAs, Co-expression networks

## Abstract

**Background:**

The amount of intramuscular fat can influence the sensory characteristics and nutritional value of beef, thus the selection of animals with adequate fat deposition is important to the consumer. There is growing knowledge about the genes and pathways that control the biological processes involved in fat deposition in muscle. MicroRNAs (miRNAs) belong to a well-conserved class of non-coding small RNAs that modulate gene expression across a range of biological functions in animal development and physiology. The aim of this study was to identify differentially expressed (DE) miRNAs, regulatory candidate genes and co-expression networks related to intramuscular fat (IMF) deposition. To achieve this, we used mRNA and miRNA expression data from the *Longissimus dorsi* muscle of 30 Nelore steers with high (H) and low (L) genomic estimated breeding values (GEBV) for IMF deposition.

**Results:**

Differential miRNA expression analysis between animals with extreme GEBV values for IMF identified six DE miRNAs (FDR 10%). Functional annotation of the target genes for these microRNAs indicated that the PPARs signaling pathway is involved with IMF deposition. Candidate regulatory genes such as SDHAF4, FBXO17, ALDOA and PKM were identified by partial correlation with information theory (PCIT), phenotypic impact factor (PIF) and regulatory impact factor (RIF) co-expression approaches from integrated miRNA-mRNA expression data. Two DE miRNAs (FDR 10%), bta-miR-143 and bta-miR-146b, which were upregulated in the Low IMF group, were correlated with regulatory candidate genes, which were functionally enriched for fatty acid oxidation GO terms. Co-expression patterns obtained by weighted correlation network analysis (WGCNA), which showed possible interaction and regulation between mRNAs and miRNAs, identified several modules related to immune system function, protein metabolism, energy metabolism and glucose catabolism according to in silico analysis performed herein.

**Conclusion:**

In this study, several genes and miRNAs were identified as candidate regulators of IMF by analyzing DE miRNAs using two different miRNA-mRNA co-expression network methods. This study contributes to the understanding of potential regulatory mechanisms of gene signaling networks involved in fat deposition processes measured in muscle. Glucose metabolism and inflammation processes were the main pathways found in silico to influence intramuscular fat deposition in beef cattle in the integrative mRNA-miRNA co-expression analysis.

**Electronic supplementary material:**

The online version of this article (10.1186/s12864-018-4514-3) contains supplementary material, which is available to authorized users.

## Background

The amount of intramuscular fat (IMF) is an important characteristic associated with juiciness and taste of beef [1]. IMF deposition is associated with size and number of adipocytes, the balance between lipogenesis and lipolysis rate in muscle and changes in catabolic activities in different species [2–5]. In humans, skeletal muscle insulin resistance can be associated with IMF deposition [6], or more recently with lipid intermediates [7]. Overall meat quality can be impacted by many factors such as nutritional program, environment, age, sex and genetics. However, little attention has been paid to the role of microRNAs in the regulation of IMF deposition in cattle.

MicroRNAs (miRNAs) are endogenous non-coding (ncRNA) ribonucleic acids (RNAs) that are approximately twenty-two nucleotides in length [8]. These molecules modulate the expression of genes at the post-transcriptional level by blocking the translation of target mRNAs [8, 9]. MiRNAs play an important role in post-transcriptional gene regulation in many tissues and are associated with the control of several important biological processes related to lipid metabolism [10]. Understanding the regulatory functions of miRNA and other small RNAs on the expression of target genes that impact lipogenesis and adipogenesis is important to identify target molecules that influence fat deposition. Several studies have been published, which demonstrate the importance of miRNAs as potential biomarkers for variations in subcutaneous adipose tissue [11–13]. However, limited information about the importance of miRNA is available for IMF [14, 15]. Once identified, biomarkers could be used in animal breeding programs to improve meat quality and animal productivity [11, 13] and potentially contribute to our understanding of insulin resistance associated with human diseases such as obesity and diabetes [16, 17].

Although RNA-seq analyses can be helpful for genomic studies and can generate lists of expressed genes in specific tissues to ultimately detect differentially expressed (DE) genes, the biological interpretation of this data is still a challenge. Network approaches that integrate data have proven useful in the identification of complex transcriptional regulation. For example, hub genes, which are highly correlated with a large number of genes, have been shown to have key regulatory roles in gene expression networks [18–20]. Thus, co-expression analysis may be more sensitive at detecting biologically interesting pathways than analysis of DE genes expression [21]. Several network approaches are available for this purpose, such as the Weighted Gene Co-expression Network Analysis (WGCNA) and the Partial Correlation with Information Theory (PCIT) methods. The WGCNA method identifies gene correlation networks, i.e. gene clusters of biological significance, from expression profiling data [22]. The PCIT method identifies differences in pairs of correlated gene expression levels to measure a gene’s differential connectivity across levels of a phenotype [23]. Utilization of both PCIT and WGCNA have enabled a better understanding of the co-regulation of mRNAs and miRNAs for different phenotypes [21, 24–27] to better comprehend the biological mechanisms and regulatory processes in lipid metabolism.

In this study, skeletal muscle microRNA and mRNA expression data from animals with different IMF deposition were integrated with two well documented systems biology methodologies [22, 23]. These analyses indicate that miRNAs play a role in IMF deposition by modulating carbohydrate, lipid and immune response metabolic pathways in skeletal muscle.

## Results

### Phenotypic and sequencing data

The genetic variance, residual variance and heritability for intramuscular fat (IMF) obtained from this population were 0.196, 0.490 and 0.29 ± 0.16, respectively, as previously published [28]. The animals were ranked using genomic estimated breeding values (GEBV) for IMF values and fifteen animals with high IMF GEBV (H) and fifteen with low IMF GEBV (L) were selected for miRNA-Seq analysis (Table 1). This strategy, to select animals with extreme GEBV, was performed because the correlation (r) between the raw IMF values (% IMF) and GEBV was high (r = 0.76) [28] and the statistical T-test showed that the GEBV averages for groups were statistically different (*p*-value = 2.2e-16).

**Table 1:** Phenotypic data for intramuscular fat percentage (IMF), genomic estimated breeding values (GEBV) and number of normalized mapped miRNA reads for all animals

Animal	IMF (%)	GEBV	Mapped Reads
High^1^	4.42	0.44	676,705.83
High^2^	4.12	0.51	722,149.21
High^3^	4.35	0.57	856,445.98
High^4^	5.02	0.47	564,721.48
High^5^	4.74	0.81	1,477,652.40
High^6^	3.99	0.51	382,812.08
High^7^	4.17	0.66	1,372,859.97
High^8^	4.95	0.59	714,291.96
High^9^	3.97	0.57	637,211.41
High^10^	4.38	0.71	628,643.15
High^11^	5.27	0.85	803,295.42
High^12^	4.35	0.61	675,159.48
High^13^	3.75	0.42	610,860.91
High^14^	2.99	0.36	327,429.32
High^15^	4.13	0.81	578,590.62
Low^1^	2.06	−0.57	681,969.11
Low^2^	1.32	− 0.77	825,926.83
Low^3^	1.35	−0.36	654,790.36
Low^4^	1.7	−0.31	510,809.28
Low^5^	1.44	−0.51	661,870.55
Low^6^	1.04	−0.33	675,033.41
Low^7^	1.58	−0.5	711,330.33
Low^8^	1.39	−0.52	421,474.63
Low^9^	1.94	−0.29	727,950.69
Low^10^	1.86	−0.24	980,827.28
Low^11^	1.38	−0.43	754,990.11
Low^12^	1.6	−0.59	655,706.88
Low^13^	1.62	−0.57	862,654.84
Low^14^	0.65	−0.22	821,821.32
Low^15^	1.69	−0.27	1,398,620.62
Mean High	4.306	0.592	735,255.28
Mean Low	1.508	−0.432	756,385.08

A total of 32 million (M) sequence reads were obtained from an Illumina MiSeq. The average number of total reads per sample was one million. The total number of mapped reads was 24 M with an average of 84% reads mapped (Table 1).

### Differentially expressed microRNAs and target genes identification

Twenty-six novel and 463 known miRNAs were identified using miRDeep2. Among all microRNAs identified, six of them were differentially expressed (DE) with a False Discovery Rate (FDR) of 10% (Table 2). Negative values of fold change indicate lower miRNA expression in animals with low IMF deposition and positive values indicate higher miRNA expression for this group. These six microRNAs targeted 2250 genes expressed in skeletal muscle based on IPA analysis (Additional file 1: Table S1). Of note, because bta-let-7f and bta-let-7a-5p belong to the same family of miRNAs and have the same seed sequence, they most likely target the same genes (Table 2).

**Table 2:** List of differentially expressed miRNAs between Low and High groups, based on genomic estimated breeding values (GEBV) for intramuscular fat, identified by miRDeep2 and the number of target genes obtained by IPA® for each miRNA

miRNA	FDR^1^	Fold Change^2^	Low GEBV^3^	High GEBV^3^	Target Genes^4^
bta-let-7f	0.04	−1.67	2617.43	3767.18	1236
bta-let-7a-5p	0.08	−1.45	1526.04	1908.20	1236
bta-miR-146b	0.08	1.55	423.46	301.78	544
bta-miR-100	0.09	1.71	1968.02	840.72	176
bta-miR-143	0.09	1.30	32,275.17	27,539.78	648
bta-miR-423-5p	0.09	−1.60	311.91	488.05	294

^1^False discovery rate adjusted p-values by Benjamini-Hochberg methodology

^2^Log2 Fold Change from low to high groups

^3^Mean normalized counts from low and high groups

^4^Target genes identified by IPA

### Enrichment analysis of target genes from the DE microRNAs

**Fig. 1:**
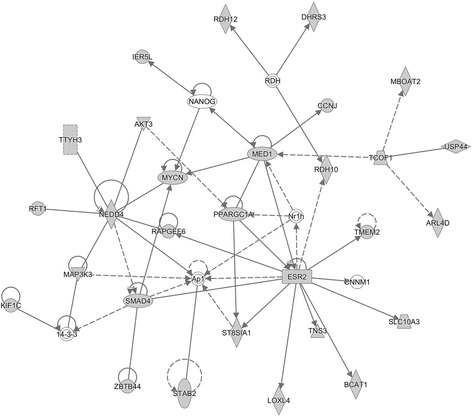
Gene network “lipid Metabolism, Small Molecule Biochemistry, Vitamin and Mineral Metabolism” identified from the DE miRNA’s target genes list generated by IPA. Grey shapes represent target genes and the white shapes are other genes of the network that are not target genes. Solid lines mean direct interaction and dashed lines an indirect interaction between genes

Functional enrichment analyses of target genes by IPA showed networks and canonical pathways related to fatty acid metabolism (Table 3). Gene networks and the principal canonical pathways are described in Additional file 2: Table S2, Additional file 3: Table S3, Additional file 4: Figures S1, S2 and S3, and Additional file 5: Figures S4, S5 and S6. The most relevant gene network was “lipid metabolism, small molecule biochemistry, vitamin and mineral metabolism” that involved genes such as *PPARGC1A*, *MYCN*, *ESR2* and *ARL4D*, that are targets of downregulated miRNAs and *MED1*, *SMAD4*, *NEDD4* and *MBOAT2*, that are targets of upregulated miRNAs in the L group (Fig. 1).

**Table 3:** List of the top gene networks and signaling pathways related with lipid metabolism identified by IPA®

Gene Networks	Target genes	P-score^1^	Signaling Pathways	Target genes	*P*-value^2^
Drug Metabolism, Lipid Metabolism, Molecular Transport	32	31	PPAR Signaling	33	1.00E-08
Lipid Metabolism, Small Molecule Biochemistry, Vitamin and Mineral Metabolism	31	30	PPARα-RXR Activation	44	3.00E-06
Gene Expression, Cell Cycle, Cancer	32	28	Adipogenesis	27	0.003

^1^P–score = −log_10_ (*p*-value)

^2^Nominal p-value

**Fig. 2:**
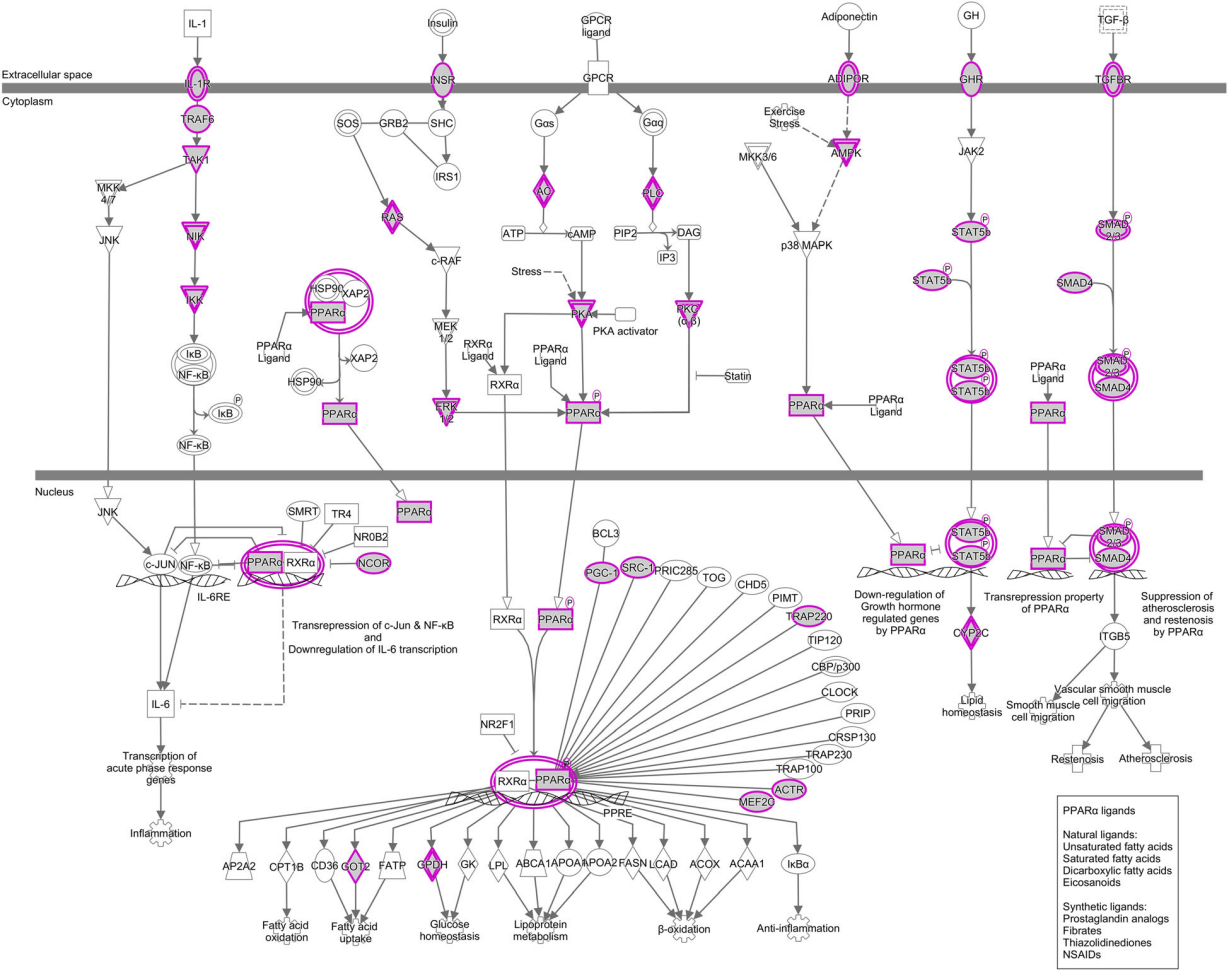
The PPARα-RXR signaling pathway is over-represented in miRNA target genes identified by IPA. The shapes highlighted in purple represent the miRNA target genes and the white shapes represent the other genes of the pathway that are not target genes

Target genes enriched for PPAR-RXR signaling pathways (i.e. lipogenesis promoting) were negatively regulated by miRNAs which were upregulated in L group. Target genes associated with fatty acid oxidation were targets of downregulated miRNAs. Some important genes for lipid metabolism present in this pathway included: *PPARa*, *PKA* and *ADIPOR2*. These genes are targets of the downregulated miRNAs bta-let-7 and bta-miR-423 (i.e. downregulated in the L group). On the other hand, *STAT5b* and *GPDH* are targets of upregulated miRNAs (bta-miR-100 and bta-miR-143) in L group (Fig. 2).

### Co-expression analysis: PCIT - differential Hubbing

After data filtering by expression in high (H) IMF and low (L) IMF groups, a list with 383 miRNAs and 14,650 genes expressed in bovine skeletal muscle were used for Partial Correlation with Information Theory (PCIT) analysis, which allowed for the identification of ten positive and negative differentially hubbed (DH) genes and miRNAs (Additional file 6: Table S4; Table 4).

**Table 4:** List of the top ten positive and negative Differentially Hubbed (DH) genes and miRNAs, when comparing High and Low groups of GEBV for IMF

ENSEMBL Gene ID	Gene Symbol	DH
Top Positive Differentially Hubbed genes		
ENSBTAG00000009084	*ATG3*	1849
ENSBTAG00000005688	*MRPS2*	1793
ENSBTAG00000008664	*EIF2B2*	1785
ENSBTAG00000012113	*HCCS*	1781
ENSBTAG00000005196	*TYW3*	1755
ENSBTAG00000001022	*AMDHD2*	1750
ENSBTAG00000010339	*ABHD11*	1736
ENSBTAG00000017941	*NSUN5*	1735
ENSBTAG00000003066	*NSA2*	1731
ENSBTAG00000001783	*FBXO17*	1730
Top Negative Differentially Hubbed genes		
ENSBTAG00000027049	*SDHAF4*	−851
ENSBTAG00000010952	*C2CD4B*	− 850
ENSBTAG00000005275	*PKIG*	− 837
ENSBTAG00000009876	*C4BPA*	−835
ENSBTAG00000011184	*FTH1*	− 828
ENSBTAG00000008895	*BPGM*	−819
-	bta-miR-24-3p	− 811
-	bta-miR-1291	− 810
ENSBTAG00000031778	*HIST1H2BD*	− 799
ENSBTAG00000038275	*CYP27C1*	−795

**Fig. 3:**
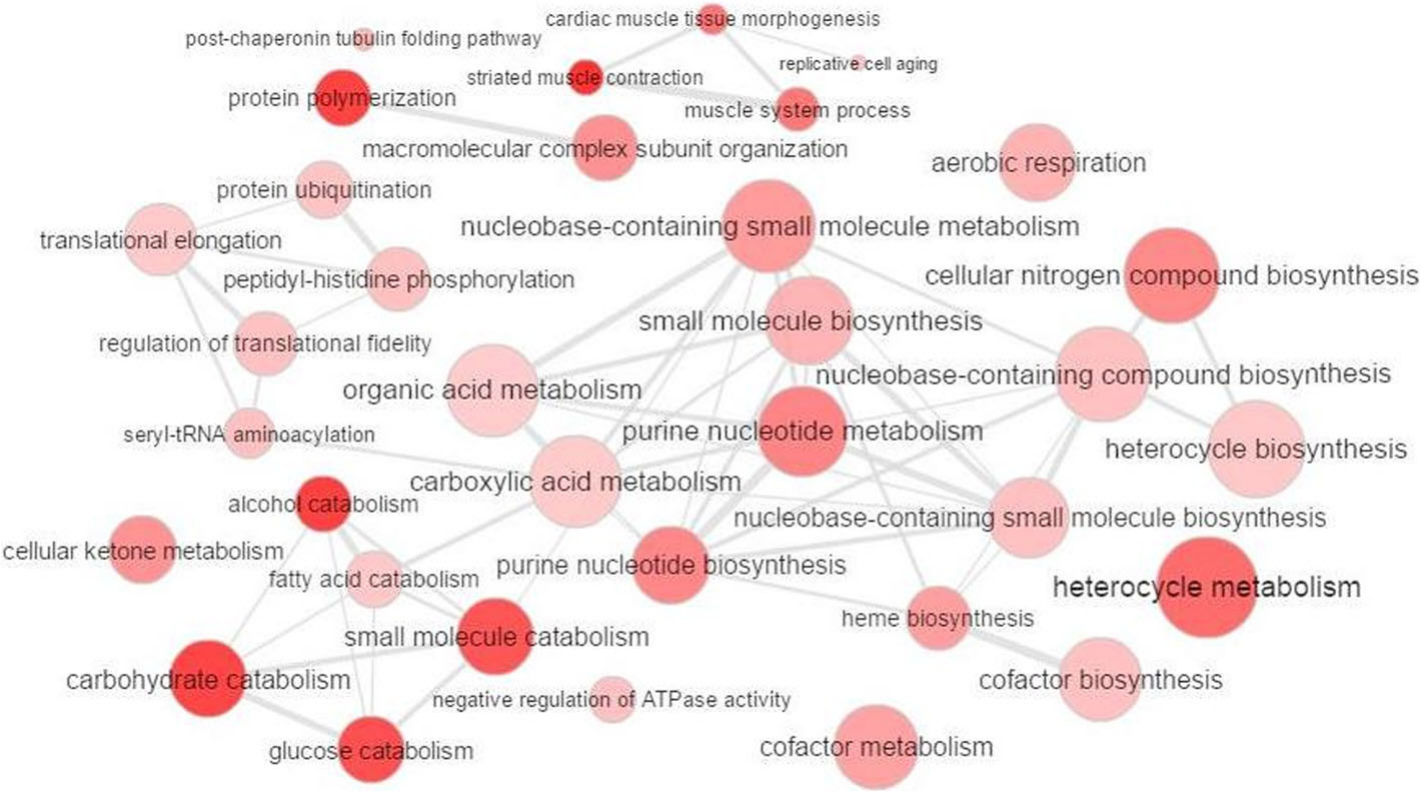
GO terms enriched from genes significantly correlated with negatively differentially hubbed (DH) genes. Bubble color indicates the user-provided *p*-value and the lower the p-value of the processes grouped in the category, the more intense is the coloring of bubbles; bubble size indicates the frequency of the GO term in the underlying GOA database. Highly similar GO terms are linked by edges in the graph, where the line width indicates the degree of similarity

**Fig. 4:**
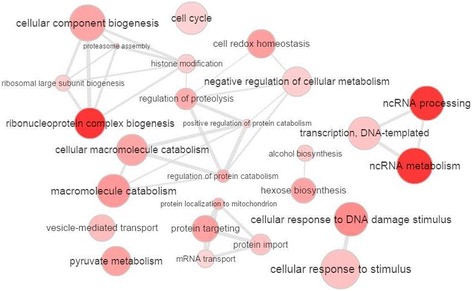
GO terms enriched from genes significantly correlated with positively differentially hubbed (DH) genes. Bubble color indicates the user-provided *p*-value and the lower the p-value of the processes grouped in the category, the more intense is the coloring of bubbles; bubble size indicates the frequency of the GO term in the underlying GOA database. Highly similar GO terms are linked by edges in the graph, where the line width indicates the degree of similarity

**Fig. 5:**
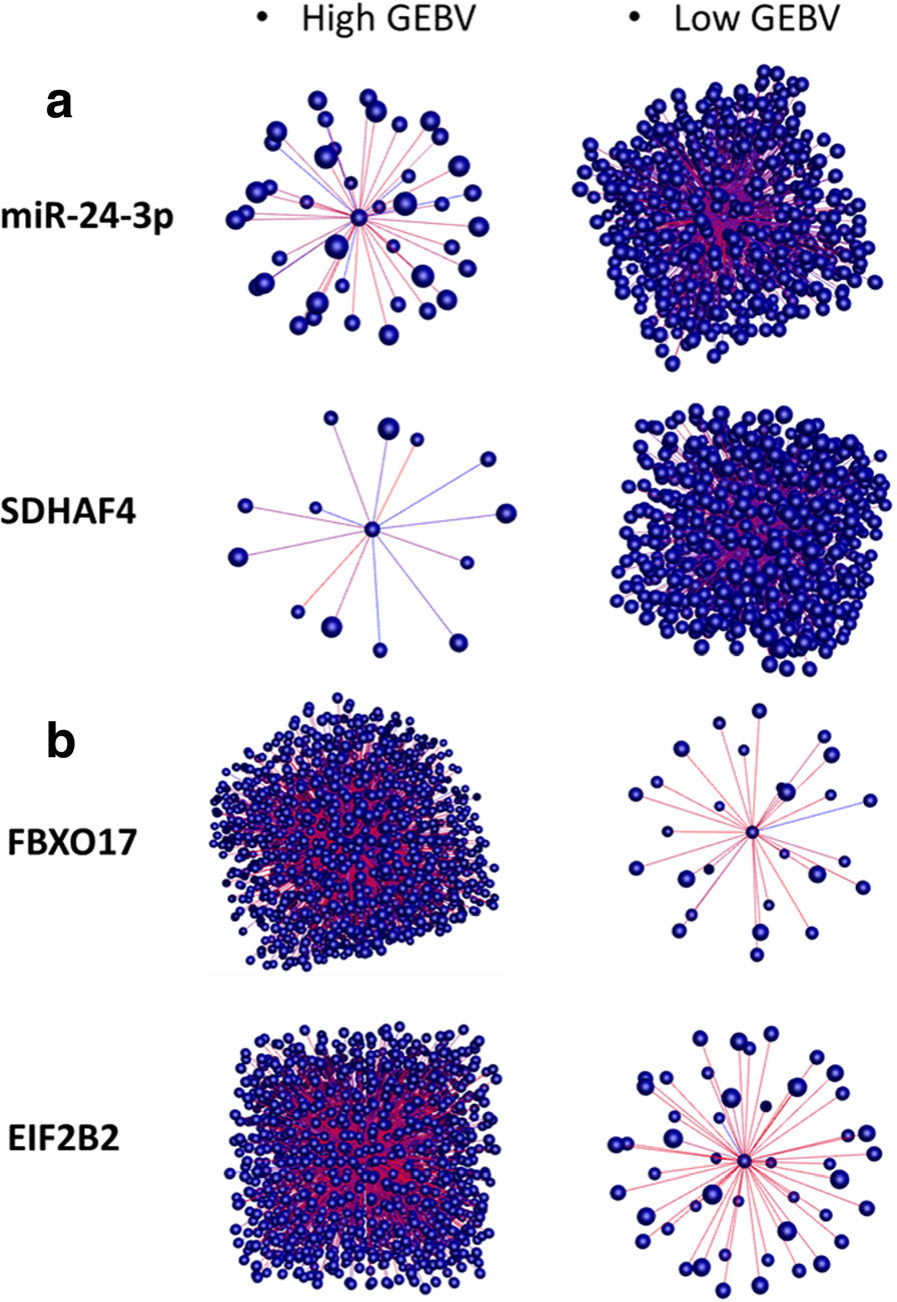
Negative (**a**) and positive (**b**) differentially hubbed (DH) genes associated with lipid metabolism between the High GEBV and Low GEBV IMF groups. The blue edges represent negative correlations between hub gene and the correlated genes while the red edges represent positive correlations

The genes with a significant correlation with DH genes were used to construct co-expression networks and identify enriched GO terms (Additional file 7: Tables S5 and S6, Additional file 8: Tables S7 and S8). The GO terms enriched among all genes correlated to the top ten negative DH genes were most related to glucose metabolism (GO ID: 6006, GO ID: 6007, GO ID: 6096) (Fig. 3) and for the top ten positive DH genes the GO terms were related to protein and mRNA metabolism (GO ID: 6364, GO ID: 6350, GO ID: 30,163, GO ID: 30,162, GO ID: 51,603) (Fig. 4). The DE miRNAs bta-miR-143 and bta-miR-146b were upregulated in the low (L) IMF group (Table 2), were positively correlated with negative DH genes, and were associated with glucose and fatty acids catabolism (Fig. 3). The most important DH genes potentially involved in the regulation of lipid metabolism and protein metabolism are shown in Table 5. The co-expression networks of top DH genes were visualized by BioLayout (Fig. 5).

**Table 5:** List of the top two differentially hubbed (DH) genes and the GO terms associated with them. The negative DH genes have higher number of connections in Low GEBV group and positive DH genes in High GEBV group

ENSEMBL Gene ID	Gene Symbol	DH	GO terms of genes correlated
Top Negative Differentially Hubbed genes			
ENSBTAG00000027049	*SDHAF4*	−851	GO ID 44275:cellular carbohydrate catabolic process
			GO ID 44282:small molecule catabolic process
			GO ID 16052:carbohydrate catabolic process
	bta-miR-24-3p	−811	GO ID 44275:cellular carbohydrate catabolic process
			GO ID 6096:glycolysis
			GO ID 6936:muscle contraction
Top Positive Differentially Hubbed genes			
ENSBTAG00000008664	*EIF2B2*	1785	GO ID 6090:pyruvate metabolic process
			GO ID 30162:regulation of proteolysis
			GO ID 6364:rRNA processing
ENSBTAG00000001783	*FBXO17*	1730	GO ID 30162:regulation of proteolysis
			GO ID 6364:rRNA processing
			GO ID 70585:protein localization in mitochondrion

### Co-expression analysis: Phenotype impact factor (PIF) and regulatory impact factor (RIF)

The PIF and RIF analyses were used to identify putative regulatory genes that may explain differences in phenotype between groups of animals, based on differences in gene expression (Additional file 9: Tables S9, S10 and S11). The most relevant genes for fatty acid metabolism found in the top RIF and PIF analyses and the GO terms associated with them are shown in Table 6. The genes with positive values for RIF 1 and 2 were *PYGM, ENO3, ATP2A1, GAPDH* and *ALDOA*, which were principally related to glucose metabolism and energy metabolism. Those with negative values of RIF were mostly miRNAs. Specifically miRNA bta-miR-143, which was identified as DE in this study (Table 1), was a miRNA with one of the most negative RIF2 value. The PIF analysis also identified *ALDOA* as a putative regulatory gene for the difference in fat deposition between H and L groups. The enrichment of GO terms was similar with those found in DH analysis (Additional file 8: Tables S7 and S8; Additional file 10: Table S12).

**Table 6:** List of the genes with the most extreme Phenotypic Impact Factor (PIF) and Regulatory Impact Factor (RIF) 1 and 2 values and the GO terms associated with them

ENSEMBL Gene ID	Gene Symbol	Score	GO terms of genes correlated/targets
Top PIF			
ENSBTAG00000012927	*ALDOA*	4.895E + 10	GO ID 30163:protein catabolic process
			GO ID 6006:glucose metabolic process
			GO ID 6091:generation of precursor metabolites and energy
Top Positive RIF1 and 2			
ENSBTAG00000012927	*ALDOA*	105.5434	GO ID 6006:glucose metabolic process
ENSBTAG00000005534	*ENO3*	4.121744	GO ID 6007:glucose catabolic process
ENSBTAG00000001032	*PYGM*	6.417047	GO ID 16052:carbohydrate catabolic process
ENSBTAG00000014731	*GAPDH*	4.403429	GO ID 22900:electron transport chain
ENSBTAG00000006541	*ATP2A1*	5.891178	GO ID 6006:glucose metabolic process
Top Negative RIF2			
ENSBTAG00000001601	*PKM*	−0.5742	GO ID 6006:glucose metabolic process
ENSBTAG00000030114	bta-miR-143	−0.74594	GO ID 6538:glutamate catabolic process
ENSBTAG00000029850	bta-miR-26b	−0.84051	GO ID 6793:phosphorus metabolic process

### Co-expression analysis: WGCNA - miRNAs correlated with mRNA modules

The weighted correlation network analysis (WGCNA) methodology was applied in two different manners, first to integrate mRNAs and miRNAs by analyzing those modules (i.e. co-expression networks) that had a negative correlation between them and second by identifying modules that are important to phenotypic variation by correlating all modules identified in WGCNA with intramuscular fat (IMF) deposition. A total of 27 mRNA modules in high (H) IMF and 44 in the low (L) IMF group were identified. Furthermore, there were 14 miRNA modules in H and 22 in L. The grey module contained all genes not included in a correlated module (Additional file 11: Tables S13 and S14; Additional file 12: Tables S15 and S16).

**Fig. 6:**
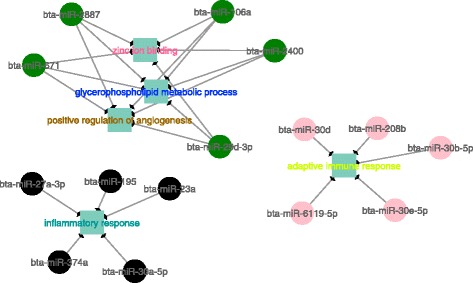
Co-expression networks showing the negative correlation among miRNAs and biological processes enriched in mRNA modules in High IMF GEBV group. Colored circles represent hub miRNAs, with higher connectivity, inside each module and squares represent the GO terms associated with each mRNA module, represented by different letter color

**Fig. 7:**
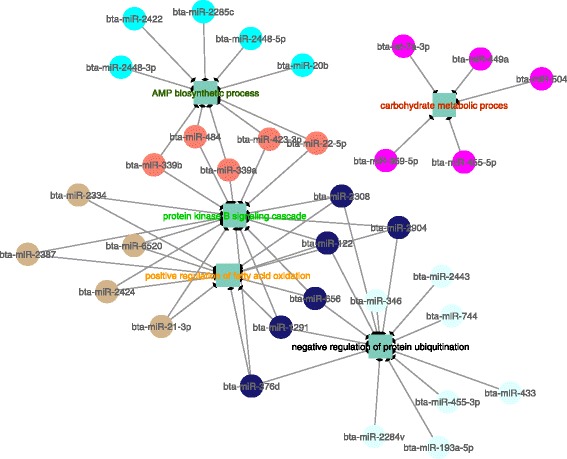
Co-expression networks show the negative correlation among miRNAs and biological processes enriched in mRNA modules in Low IMF GEBV group. Colored circles represent hub miRNAs, with higher connectivity, inside each module and squares represent the GO terms associated with each mRNA module, represented by different letter color

After correlating all miRNA and mRNA modules with one another, those modules that were negatively correlated with one another were investigated further. Among all correlated modules, three miRNA modules were negatively correlated with five mRNA modules in the H group, while six miRNA modules were negatively correlated with seven mRNA modules in the L group (*p*-value > 0.05; Additional file 13: Table S17). The genes that composed each mRNA module were significantly over enriched for GO terms related to lipid metabolism (adj. p-value < 0.1; Additional file 14: Tables S18 and S19). These lipid metabolism GO terms that were enriched from mRNA modules were then used to construct mRNA-miRNA co-expressed networks for both groups H and L (Figs. 6 and 7). The miRNAs modules were enriched for GO terms based on the hub miRNA target genes of each module (Additional file 14: Tables S20 and S21).

The WGCNA results indicated that the cyan and lightgreen mRNA modules in the high (H) IMF group were enriched for inflammatory response and adaptive immune response (Additional file 14: Table S18). They were also negatively correlated with the black and pink miRNA modules (Fig. 6), whose target genes were associated with carboxylic acid transport and positive regulation of leukocyte migration (Additional file 14: Table S20). Unlike the high (H) group, the low (L) IMF group contained multiple miRNA modules that were negatively correlated with the same mRNA module (Additional file 13: Table S17, Fig. 7). The target genes of the most connected miRNA module (midnightblue) in the L group were associated with metal ion homeostasis and regulation of interleukin-1 beta (*IL-1B*) production (Additional file 14: Table S21). This module was negatively correlated with four mRNA modules (green, orange, yellowgreen and black) (Fig. 7). These four mRNA modules were enriched for protein kinase B (*PKB*) signaling cascade, positive regulation of fatty acid oxidation and negative regulation of protein ubiquitination (Additional file 14: Table S19).

The hub miRNAs in the green module from the high IMF group (e.g. bta-miR-106a, bta-miR-671) (Fig. 6) and midnightblue module from the low IMF group (e.g. bta-miR-122, bta-miR-1291) (Fig.7) identified in the WGCNA, were also found correlated with the DH genes in the PCIT analysis (Additional file 7: Tables S5 and S6). Additionally, the miR-1291 was both a hub in the WGCNA midnightblue module from low (L) IMF group (Fig. 7) and a negatively DH gene (Table 4). These results indicate that WGCNA and PCIT not only identify similar biological processes associated with fat deposition, but they can also identify the same miRNAs in co-expression networks.

### Co-expression analysis: WGCNA – Modules correlated with phenotype

Besides the integration of mRNA and miRNA data, the correlation of modules with the phenotype (high or low IMF deposition) was also performed (Additional file 15: Figure S7; Additional file 16: Figure S8). Three mRNA modules in the high (H) IMF group and two in the low (L) IMF group, and three miRNA modules both the H and L groups were correlated with IMF (Table 7). In the H group, the black miRNA module and the cyan mRNA module were negatively correlated with each other (Additional file 13: Table S17).

**Table 7:** GO term enrichment of modules significantly correlated with the IMF trait, for mRNAs and miRNAs in the High (H) and Low (L) groups

Group	module	corr	p-value	#molecules	FDR	GO terms
microRNAs modules						
H	black	0.7	0.007	21	0.045	GO ID 46942:carboxylic acid transport
	yellow	0.5	0.09	25	0.041	GO ID 6417:regulation of translation
	turquoise	−0.5	0.07	105	0.064	GO ID 19377:glycolipid catabolic process
L	greenyellow	0.5	0.04	13	0.007	GO ID 6629:lipid metabolic process
	lightgreen	0.6	0.02	7	0.0001	GO ID 55074:calcium ion homeostasis
	purple	−0.5	0.07	14	0.028	GO ID 19915:lipid storage
mRNAs modules						
H	darkorange	0.5	0.09	63	0.052	GO ID 42816:vitamin B6 metabolic process
	cyan	−0.5	0.08	187	0.01	GO ID 6954:inflammatory response
	darkgrey	0.5	0.08	83	0.002	GO ID 4117:calmodulin-dependent cyclic- nucleotide phosphodiesterase activity
L	plum1	−0.5	0.06	63	0.053	GO ID 19955:cytokine binding
	magenta	−0.5	0.03	474	0.093	GO ID 2385:mucosal immune response

The enrichment analysis results of modules correlated with IMF are presented in Table 7. The table shows the biological processes downregulated by miRNAs modules correlated with phenotype and the biological processes associated with mRNAs modules correlated with phenotype in both groups. The GO terms associated with each mRNA and miRNA module that was significantly correlated with IMF are presented in Additional file 17: Tables S22, S23, S24 and S25.

The hub miRNAs in modules that were correlated with intramuscular fat (IMF) in both H and L groups downregulate processes related with lipid metabolism. The mRNA modules that were correlated with IMF in both groups were enriched for inflammatory response and immune system GO terms (Table 7).

## Discussion

The regulation of lipid oxidation and biosynthesis is under strong feedback control in order to maintain homeostasis [29]. Intramuscular fat deposition in cattle is of economic importance and has been studied in several breeds, including Nelore under different nutritional conditions [30]. However, there is still limited knowledge on the molecular processes, in particular regarding microRNA involvement in the control of this trait [31]. It is important to remember that samples collected in this study are from the *Longissimus dorsi* (LD) muscle and thus contains myofibers and other cell types such as adipocytes, fibroblast, blood and nerve cells. Thus, the gene expression profile obtained can be influenced by the proportion of the different cell types in the sample and/or by genes being differentially expressed in a specific cell type.

### Networks enriched for lipid and carbohydrate metabolism

Although research studying the influence of miRNAs on metabolism has been recently published [32], there has been a rapid growth in the number of identified miRNAs that are involved in the regulation of genes and signaling molecules responsible for maintaining lipid homeostasis [26, 33].

The comparison of miRNA expression between animals with different genetic potential for intramuscular fat (IMF) deposition resulted in the identification of six differentially expressed (DE) miRNAs involved in fatty acid metabolism and lipid deposition. The miR-423 and let-7 family, upregulated in the high (H) IMF group, have been previously reported to be associated with obesity in humans and disorders in glucose metabolism in mice [16, 34] and have been implicated as possible biomarkers for risk of obesity [16, 34]. The miRNAs upregulated in the low (L) IMF group, miR-100, miR-146 and miR-143, have been reported to control aspects of adipogenesis in humans [35–38]. By in vitro analysis Chen et al. [35] suggested that overexpression of mir-143 in adipose-derived stem cells (ADSCs) in rats could promote or inhibit adipogenesis by regulation of *MAPK* signaling pathway depending on the stage of development. Interestingly, they found that upregulation of miR-143 expression in early stages of adipogenesis blocks adipocyte differentiation, but when it happens later induces clonal expansion of adipose tissue.

The enrichment analysis of DE miRNAs’ target genes revealed that the *PPAR* pathway was overrepresented in IPA analysis (Additional file 5: Figures S4, S5 and S6). Furthermore, many important target genes related to lipid metabolism were present in the gene networks identified by IPA (Table 3 and Additional file 4: Figures S1, S2 and S3). Among them, PPARs were found, which are a class of ligand-activated transcription factors that have a well-known influence on lipid metabolism and glucose homeostasis [39–43]. The target genes of miRNAs downregulated in the low (L) IMF group were enriched for the GO term fatty acid oxidation involved in *PPARa* pathway. On the other hand, based on IPA enrichment results, miRNAs upregulated in this group would downregulate genes of lipogenesis and adipogenesis.

In a previous comparison of gene expression profile between Angus (higher IMF deposition capacity) and Nelore, Martins et al. (2015) [30] did not observed a difference in *PPARγ* mRNA expression; however, a higher amount of *PPARγ* protein was detected by western blot. This result highlights the limitation of individual mRNA gene expression analysis and shows the importance of a systems biology approach, in which co-expression analysis can indicate a modulation in a pathway even without detectable difference in individual gene expression.

To gain additional insights into the pathways impacted by miRNAs, we performed co-expression analysis by integration of the miRNA and mRNA data. The PCIT analysis revealed that the top negatively differentially hubbed (DH) genes, which had more connections in the low (L) IMF group, were correlated with genes associated mostly with carbohydrate metabolism (Table 5). The DH genes that may be the most relevant for IMF were *SDHAF4* and bta-miR-24. *SDHAF4* is important for the assembly of succinate dehydrogenase and plays a role in ATP synthesis by the electron transport chain. Both *SDHAF4* and bta-miR-24 are associated with carbohydrate metabolism and could indicate that a change in myofiber type is associated with IMF. However, in our previous study in which we measured DE genes associated with IMF in these same animals [28], there was no significant difference in myosin heavy chain (*MyHC*) isoform expression. Interestingly, miR-24 has been shown to negatively regulate adipocyte differentiation and hepatic lipid accumulation in mice [44, 45].

The candidate regulatory genes identified by PIF and RIF that negatively regulate intramuscular fat (IMF) deposition were *PKM,* bta-miR-143 and bta-miR-26b. *PKM* is associated with glucose metabolism, while bta-miR-26b was related to control cholesterol efflux and lipogenesis in mice [46, 47] (Table 6). The target genes of bta-miR-143 were enriched for glutamate catabolism. Glutamate is a key component in cellular metabolism, and it is related to biosynthesis of lipids, because it is utilized in the citric acid cycle to produce ATP through α-ketoglutarate [48]. MiR-143 downregulates this process by blocking excess ATP production that could induce storage of lipids instead of undergoing lipid degradation. This co-expression analysis reaffirms the importance of the bta-miR-143 in control of fat deposition.

WGCNA revealed that the mRNA module in the high (H) IMF group that was positively correlated with IMF deposition (darkorange) was enriched for vitamin B6 metabolic process (Table 7), which is indirectly related to lipogenesis. Several enzyme reactions involved in fatty acid metabolism require vitamin B-6 as a coenzyme, such as the biosynthesis of sphingolipids [49], which are a class of lipids that are components of cell membranes. Moreover, the black and yellow miRNA modules in the high (H) IMF group were positively correlated with IMF deposition. The target genes for the miRNA in these modules are associated with carboxylic acid transport and regulation of translation (Table 7). Fatty acids are carboxylic acids and their transport into the mitochondria leads to activation of β-oxidation to produce energy. This indicates that miRNAs associated with high fat deposition are downregulating biological processes such as transport and catabolism of fatty acids, while miRNAs negatively associated with higher fat deposition downregulate glycolipid degradation. Interestingly in the high (H) IMF group, the black miRNA module and cyan mRNA module were negatively correlated with each other (Additional file 13: Table S17) and both were differently correlated with IMF, positively and negatively, respectively (Table 7). These miRNAs were associated with downregulation of fatty acid transport, while the mRNAs were associated with inflammation.

In the low (L) IMF group, the greenyellow and lightgreen miRNA modules were positively correlated with IMF. The miRNA in these modules downregulate genes enriched for lipid metabolic process and calcium ion homeostasis, respectively (Table 7). Calcium (Ca) participates in many signaling networks that contribute to modulation of enzyme function, including Ca-sensitive enzymes involved in lipolysis and lipogenesis [50]. The purple miRNA module was negatively correlated with IMF. Most of the miRNAs in this module were expressed at lower levels in lean animals. The target genes of these miRNA were associated with lipid storage. Overall, the miRNAs in co-expression networks associated with low IMF were related to lipid metabolism, lipolysis, lipogenesis and lipid storage.

### Networks related to immune system and inflammatory response

It is known that lipid accumulation in obesity activates the immune system which leads to an inflammatory state due to secretion of proinflammatory molecules by adipocytes [51]. Genes associated with inflammatory response were identified as target genes of DE miRNAs that were upregulated in the low (L) IMF group, which was enriched for the *PPAR-RXR* signaling pathway (Fig. 2). These genes mediate signal transduction from members of the interleukin-1 (*IL-1*) family. *IL-1,* which is regulated by *PPARa*, can induce and regulate a network of proinflammatory cytokines that initiate inflammatory responses [52]. Using the same population of animals as utilized here, Cesar et al. [28] previously reported that DE genes were associated with inflammatory response.

The WGCNA results of co-expressed mRNA-miRNA modules in the high (H) IMF group (Fig. 6) were enriched for GO terms associated with inflammatory response and adaptive immune response. These same modules were indirectly correlated with carboxylic acid transport and leukocyte migration (Additional file 13: Table S17). Adaptive immune cells have been reported to be increased in obese mice and humans, which can trigger a sequence of proinflammatory reactions and could be associated with impaired glucose tolerance and insulin sensitivity [53–56]. This indicates that mRNA and miRNA co-expression networks constructed for animals with differences in lipid accumulation (high IMF group) may be involved in pathways that regulate the immune system and inflammation and are correlated with lipid metabolism.

## Conclusion

A combination of DE and co-expression based analyses indicate that lipid metabolism, glucose metabolism and inflammatory response are the main biological processes associated with IMF deposition in Nelore cattle. The miRNAs identified in this study were not only associated with extreme intramuscular fat deposition levels, but also participate in co-expression networks that may affect mRNA expression and metabolic pathways modulating fat deposition. Furthermore, both co-expression approaches could construct similar miRNAs networks that were correlated with genes and pathways important for IMF accumulation. This study allowed us to better understand the potential roles of miRNA regulation and interaction in fat deposition and revealed new candidate regulatory genes and miRNAs associated with IMF.

## Methods

### Animals and phenotypic data

Genotypic and phenotypic data were collected on 310 Nelore steers sired by 34 unrelated sires that represent the main breeding lineages in Brazilian Nelore from an experimental breeding herd from EMBRAPA between 2009 and 2011 [57]. The animals were raised in feedlots under identical nutrition and handling conditions until slaughter at an average age of 25 months. Samples from *Longissimus dorsi* (LD) muscle located between the 12th and 13th ribs were collected at two time points: at slaughter for RNA sequencing analysis, and 24 h after slaughter for the intramuscular fat (IMF) deposition measurement as described below [28].

Approximately 100 g of muscle were lyophilized and ground to measure IMF deposition using an Ankom XT20 extractor and the AOCS procedure (official Procedure Am 5–04) as described Cesar et al. [57]. Animals with extreme values for intramuscular fat (IMF) deposition were selected based on their genomic estimated breeding values (GEBV) [57]. GEBV was predicted by Genomic Best Linear Unbiased Prediction (GBLUP) methodology, which was conducted using ASREML software [58]. A group of 30 animals were selected (fifteen with high IMF GEBV values and fifteen with low IMF GEBV values) for mRNA and miRNA analyses.

### RNA extraction and RNA-sequencing

Total RNA was isolated from 100 mg of LD muscle samples from 30 steers with extreme GEBV values. The extraction of total RNA was performed using the Trizol reagent (Invitrogen) according to the protocol described by Chomczynski and Sacchi [59]. After extraction, total RNA was quantified by spectrophotometer (NanoDrop 200 - Thermo Scientific. Wilmington. Delaware, USA). The integrity of the RNA was verified by size separation on a 1% agarose gel and analysis on a Bioanalyzer 2100 (Agilent Technologies - Santa Clara, CA, USA) with the RNA 6000 Nano kit. All samples had an RNA integrity number (RIN) greater than or equal to 8. Then samples were diluted to a final concentration of 200 ng/μL. Sequencing libraries were generated with the TruSeq® smallRNA Sample Preparation kit (Illumina - San Diego, USA). The concentration of the cDNA libraries was determined with the KAPA Library Quantification Kit (KAPA Biosystems) and then samples were sequenced on a Miseq machine (Illumina), using MiSeq Reagent Kit v3 (150 cycles), generating around 1 million reads/sample.

### Reads filtering and miRNAs identification

After sequencing, data quality was evaluated with FastQC [60] and filtered by Phred score quality using FASTX-Toolkit [61] software, where the minimum quality Phred score was 28. Then, the miRDeep2 [62] program was used to identify and quantify miRNAs, using the default parameters. The sequences were mapped against the bovine reference genome *Bos taurus* UMD 3.1 and compared with miRBase database (v. 21) [63].

### Differentially expressed miRNAs

In order to identify differentially expressed (DE) miRNAs between the L and H groups, the total count data of each miRNA was analyzed with the DESeq2 package [64], using a statistical model that fitted contemporary group (animal origin and year that the animal enter the experiment) as a categorical fixed effect and age at slaughter of an animal as a covariate. To remove variation due to the preparation of sequencing libraries, the expression data were normalized by library size, as described in the manual of the DESeq2 package [64]. The Benjamini-Hochberg (BH) [65] methodology was used to control the False Discovery Rate (FDR) of DE at 10%.

### Identification of miRNA target genes and enrichment analysis of DE miRNAs

The miRNA target genes were obtained from the MicroRNA Target Filter tool of QIAGEN’s Ingenuity Pathway Analysis (IPA®, Redwood City-CA) that uses TargetScan, miRecords and TarBase as the miRNA target genes databases. The IPA® uses information from predicted targets of mammalian microRNAs, based on the fact that target sites are usually conserved because miRNAs are highly evolutionarily conserved [66–69]. After this first approach, to obtain the target genes by IPA, we selected only those target genes that were expressed in our animals, using the data generated by RNA-seq of skeletal muscle samples of the same animals from previous study [28]. The functional enrichment of target genes was also performed by IPA software to identify enriched metabolic pathways and gene networks associated with lipid metabolism.

### PCIT and differential hubbing (DH) network analysis

To improve the functional annotation of miRNA and mRNA interactions in a systems biology context, the Partial Correlation with Information Theory (PCIT) analyses [70, 71] were conducted on the combined list of miRNAs (383) and mRNAs (14,650) after normalization of expression level by DESeq2. The miRNAs and mRNAs were filtered to select only those expressed in animals in both H and L IMF groups. The mRNA expression data utilized in this study was previously published by Cesar et al. [28].

PCIT was used to evaluate the specific behavior or co-expression between all miRNAs and genes and from this information, differential connectivity or hubbing (DH) [71] was calculated. Differential hubbing is the difference in the number of significant partial correlations (connections) a gene has between two different treatments, in this case compared between H and L groups and filtering those correlations higher than 0.9. BioLayout Express3D [72] software was used to visualize gene networks.

### PIF and RIF analysis

To identify putative candidate regulators responsible for the differences observed in phenotypes, the Phenotypic Impact Factor (PIF) and Regulatory Impact Factor (RIF) approaches were performed [23, 70, 73]. PIF gives a ‘weight’ for the contribution and importance of genes to the differences involved between phenotypes, based exclusively on their numerical properties. RIF is based on differences in the regulator’s correlations and it represents the relative importance of genes/miRNAs on the phenotypically relevant part of the network. The RIF1 value is based largely on changes in correlation between two treatments levels (i.e. differential wiring). The RIF2 value allows genes to be ranked as potential regulators based on the expression changes of a regulator and how it can affect the expression of other genes in the network due to treatment differences [23].

### WGCNA

The same list of genes and miRNAs used in the PCIT analysis was utilized to run the R package WGCNA [22]. This analysis constructs clusters of highly correlated genes and miRNAs in modules and allows the correlation of them to each other and to a trait (i.e. intramuscular fat (IMF) deposition). In contrast to the analysis performed with PCIT, the WGCNA was done separately for genes and then for miRNAs.

#### Modules of mRNA

To construct clusters of genes, pair-wise Pearson correlation coefficients were first calculated between all expressed transcripts to generate a signed similarity. To emphasize (weight) stronger correlations and punish weaker correlations, the signed similarity matrix was then raised to the lowest power β that approximated a scale-free network topology (R^2^ > 0.90) to generate an adjacency matrix [74]. The β’s used to construct the mRNA modules from the L and H IMF group’s expression data were 12 and 8, respectively (Additional file 18: Figure S9). The topological overlap distance calculated from the adjacency matrix is then clustered with the average linkage hierarchical clustering. The default minimum cluster merge height of 0.25 was retained. The clusters created by WGCNA are called modules, and the minimum number of genes in a module was set to 30. Each module represents a group of highly interconnected genes with similar expression profiles across the samples and the expression profile pattern is distinct from those of other modules [22]. Modules were named by a conventional color scheme and genes not classified in a correlated module were grouped in the grey module. After modules were defined, the module Eigengene (MEs) values were calculated. The Eigengene of a module is defined as the eigenvector associated with the first principal component of the expression matrix representing the expression profile of all genes within a given module [22, 75].

#### Modules of miRNA

The steps for constructing miRNA co-expression modules were as described above. After generating the signed similarity matrix, a power β value was chosen to generate the adjacency matrix, the β’s used to construct the miRNA modules were 9 for the L and 4 for the H IMF groups (Additional file 19: Figure S10). The topological overlap distance was calculated and a minimum module size of five miRNAs was chosen. Five was chosen as the minimum module size for the miRNAs due to the smaller size of the miRNA transcriptome relative to the mRNA transcriptome [22, 75].

### Correlation between mRNA and miRNA modules

An integrative analysis was performed correlating the ME of miRNAs with the ME of mRNAs, for each group. Those modules with a negative correlation higher than − 0.4 with a *p*-value < 0.05 were used for enrichment analysis. The co-expression networks among hub miRNAs, representing the whole module, and the GO terms of mRNAs inside the correlated modules were constructed in Cytoscape v.3.3.0 0 [76].

### Correlation of modules with phenotype

Using the ME, the Module-Trait relationships were estimated by calculating the Pearson’s correlations between the ME and the animals’ phenotypic information (i.e. % IMF) to select potential biologically interesting modules that could explain the phenotypic differences between groups. To avoid losing information and expand biological response, we set a p-value threshold of correlation of 0.1 to select modules correlated with trait.

### MiRNA target gene identification and enrichment analysis of co-expression data

The general gene enrichment of GO terms for biological processes was made using BiNGO (Biological Networks Gene Ontology), tool for Cytoscape v.3.3.0 [76] and REVIGO [77] to visualize clusters of GO terms. The Benjamini-Hochberg (BH) [65] methodology was used as a multiple testing correction to control the False Discovery Rate (FDR) at 10%. For miRNAs, the combined results from miRanda and TargetScan approaches were used to identify the target genes and these genes were also filtered by skeletal muscle RNA-seq data of previous study [28] to do the enrichment. MiRanda is a method for target site identification from sequence information [78]. It compares the miRNAs complementarity to 3’UTR regions of genome. Using a perl script we generated a fasta file with all 3’UTR regions of bovine genome (UMD 3.1) from ENSEMBL to use as input in miRanda. The TargetScan was performed for mammals and customized by species (cow/*Bos taurus*) (http://www.targetscan.org/vert_70/).

The enrichment of miRNA modules identified by WGCNA was conducted using the target genes information of specific hub miRNAs in each miRNA module. In this case the hub miRNAs were those with the highest Modular Membership (MM) value for the module, which means that these miRNAs have higher connectivity inside the module and are probably more informative [18].

## Additional files

Additional file 1: Table S1. Full list of DE miRNAs target genes. The table contains the Fold change (FC) of each DE miRNA and the ENSEMBL and gene symbol of their target genes. (XLSX 101 kb)

Additional file 2: Table S2. Full list of gene networks constructed by IPA using the miRNA’s target genes list. The table contains the gene symbol gene symbol of all genes inside network, the number of target genes inside each network and diseases and functions that network could participate. (XLSX 14 kb)

Additional file 3: Table S3. List of most relevant canonical pathways enriched by IPA using the miRNA’s target genes list. The table contains the name of pathway and the target genes that are present in each pathway. (XLSX 19 kb)

Additional file 4: Figures S1, S2 and S3. Gene networks most relevant for lipid metabolism that were constructed by IPA using the miRNA’s target genes list. Grey shapes represent target genes and the white shapes are other genes of the network that are not target genes. (PDF 915 kb)

Additional file 5: Figures S4, S5 and S6. The most relevant pathways enriched by IPA using the miRNA’s target genes list. The shapes highlighted in purple represent the miRNAs target genes and the white shapes represent the other genes of the pathway that are not target genes. (PDF 3964 kb)

Additional file 6: Tables S4. Full list of differential hubbing (DH) values of all genes and miRNAs used in PCIT analysis. The table contains the ENSEMBL and gene symbol of each gene. (XLSX 441 kb)

Additional file 7: Tables S5 and S6. Full list of genes significantly correlated with differential hubbed (DH) genes: genes correlated with positively DH genes (Table S5) and negatively DH genes (Table S6). The table contains the ENSEMBL of genes and the correlation value of each gene. (XLSX 745 kb)

Additional file 8: Tables S7 and S8. Gene ontology (GO) enrichment analysis by Cytoscape of genes significantly correlated with differentially hubbed (DH) genes: negative DH genes (Table S7) and positive DH genes (Table S8). The table contains gene symbol and ENSEMBL of each DH gene, gene ontology term ID, *p*-value and adjusted p-value of GO enrichments, the description of each GO term and the genes present in each enriched biological process. (XLSX 45 kb)

Additional file 9: Tables S9, S10 and S11. Full list of PIF values and adjusted *p*-values of all genes in the dataset (Table S9), RIF1 score (Table S10) and RIF2 score (Table S11). (XLSX 1975 kb)

Additional file 10: Table S12. Gene ontology (GO) enrichment analysis by Cytoscape of genes highly correlated with top PIF and RIF genes and miRNAs. The table contains gene symbol and ENSEMBL of each top gene, gene ontology term ID, *p*-value and adjusted *p*-value of GO enrichments, the description of each GO term and the genes present in each enriched biological process. (XLSX 76 kb)

Additional file 11: Tables S13 and S14. Full list of all mRNA modules and module membership (MM) values for each gene in dataset: modules constructed in the High intramuscular fat (IMF) group (Table S13), modules constructed in the Low IMF group (Table S14). (XLSX 18066 kb)

Additional file 12: Tables S15 and S16. Full list of all miRNA modules and module membership (MM) values for each miRNA in dataset: modules constructed in the High intramuscular fat (IMF) group (Table S15), modules constructed in the Low IMF group (Table S16). (XLSX 260 kb)

Additional file 13: Table S17. List of miRNA modules negatively correlated with mRNA modules and the GO terms associated with them in High IMF (H) and Low IMF (L) groups. The table contains the module’s name, the correlation value, p-value of correlation, number of miRNAs and mRNAs in each module and the GO term enriched for each module. (XLSX 11 kb)

Additional file 14: Tables S18, S19, S20 and S21. Gene ontology (GO) enrichment analysis by Cytoscape of genes in mRNA modules negatively correlated with miRNA modules: enrichment of genes from mRNA modules constructed in the High intramuscular fat (IMF) group (Table S18) and in the Low IMF group (Table S19). Gene ontology enrichment analysis by Cytoscape of target genes of hub miRNAs in modules negatively correlated with mRNA modules: enrichment of target genes of miRNA modules constructed in the High IMF group (Table S20) and in the Low IMF group (Table S21). The table contains the module’s name, gene ontology ID, p-value and adjusted p-value of GO, the description of them and the genes present in each biological process enriched. (XLSX 71 kb)

Additional file 15: Figure S7. Correlation between the mRNA modules and IMF. A: modules identified in the High IMF GEBV (H) group. B: modules identified in the Low IMF GEBV (L) group. Modules with intense red color have a higher correlation (close to + 1) and those with intense green color have a more negative correlation (close to − 1). (PDF 173 kb)

Additional file 16: Figure S8. Correlation between miRNA modules and intramuscular fat (IMF). A: modules identified in the High IMF GEBV (H) group. B: modules identified in the Low IMF GEBV (L) group. Modules with intense red color have a higher positive correlation (close to + 1) and those with an intense green color have a more negative correlation (close to − 1). (PDF 118 kb)

Additional file 17: Tables S22, S23, S24 and S25. Gene ontology (GO) enrichment analysis by Cytoscape of genes in mRNA modules correlated with the intramuscular fat (IMF) trait: enrichment of genes from mRNA modules correlated with High IMF (Table S22) and Low IMF groups (Table S23). Gene ontology enrichment analysis by Cytoscape of target genes of hub miRNAs in modules correlated with the IMF trait: enrichment of target genes of miRNA modules correlated with High intramuscular fat (Table S24) and Low IMF groups (Table S25). The table contains module’s name, gene ontology ID, p-value and adjusted p-value of GO, the description of them and the genes present in each biological process enriched. (XLSX 98 kb)

Additional file 18: Figure S9. Scale free topology model and mean connectivity of the mRNA networks based on the power value β. (A) H group. (B) L group. (PDF 92 kb)

Additional file 19: Figure S10. Scale free topology model and mean connectivity of the miRNA networks based on the power value β. (A) H group. (B) L group. (PDF 85 kb)

## Abbreviations

BH: Benjamin-Hochberg
DE: Differentially expressed
DH: Differentially hubbed
FC: Fold Change
FDR: False discovery rate
GBLUP: Genomic best linear unbiased prediction
GEBV: Genomic estimated breeding values
GO: Gene ontology
GOA: Gene ontology annotation
H: High IMF group
IMF: Intramuscular fat
IPA: Ingenuity pathways analysis
L: Low IMF group
LD: Longissimus dorsi
ME: Module eigengene
MM: Modular membership
ncRNA: non-coding RNA
PCIT: Partial correlation with information theory
PIF: Phenotypic impact factor
RIF: Regulatory impact factor
RNA: Ribonucleic acid
WGCNA: Weighted correlation network analysis
