# Supplementary figures and images for: Integrative analysis of microRNAs and mRNAs revealed regulation of composition and metabolism in Nelore cattle
Source: BMC Genomics. 2018 Feb 7;19:126. doi: 10.1186/s12864-018-4514-3 (PMC5804041; doi:10.1186/s12864-018-4514-3)

(1) Drug Metabolism, Lipid Metabolism, Molecular Transport

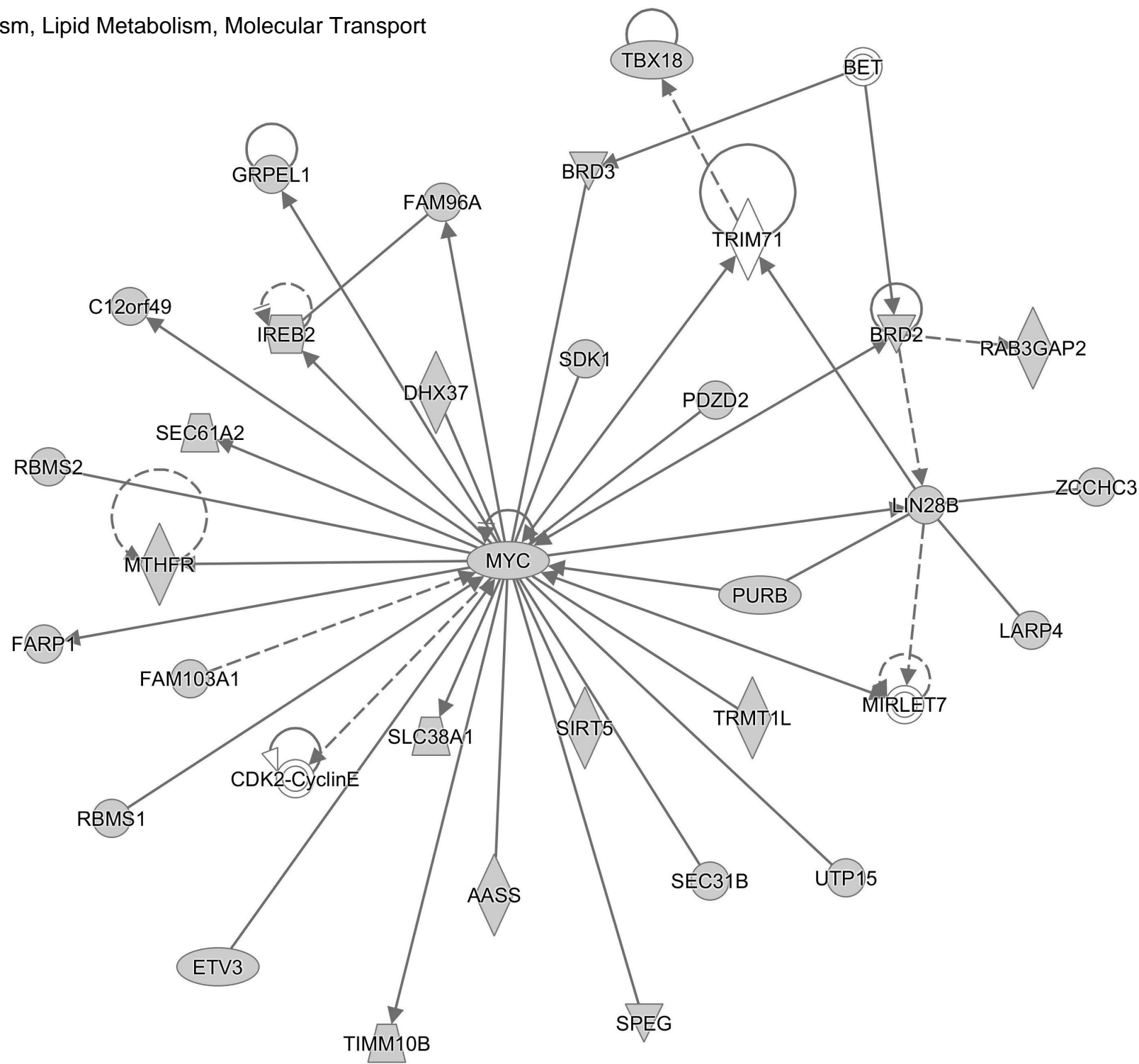

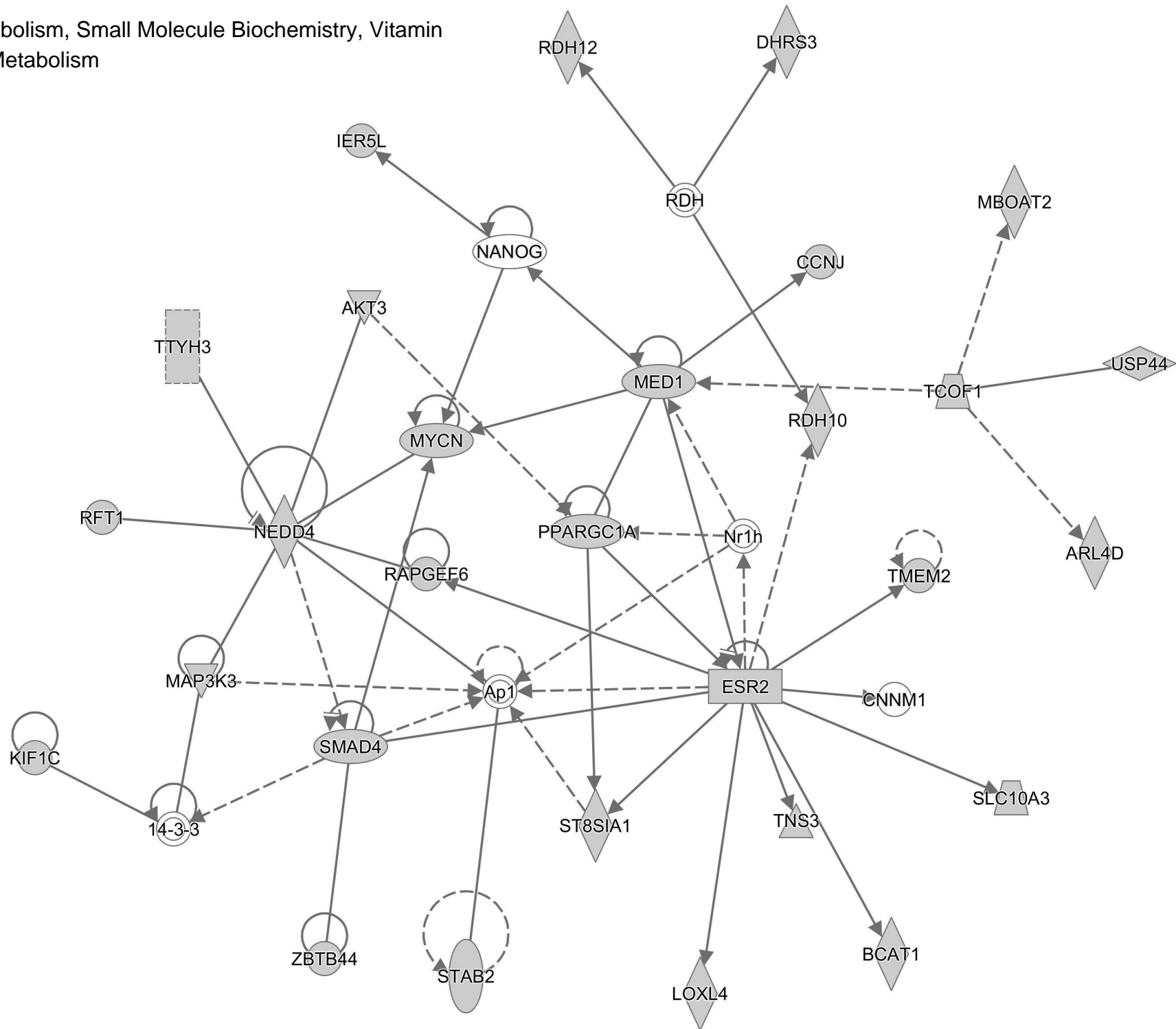

(3) Gene Expression, Cell Cycle, Cancer

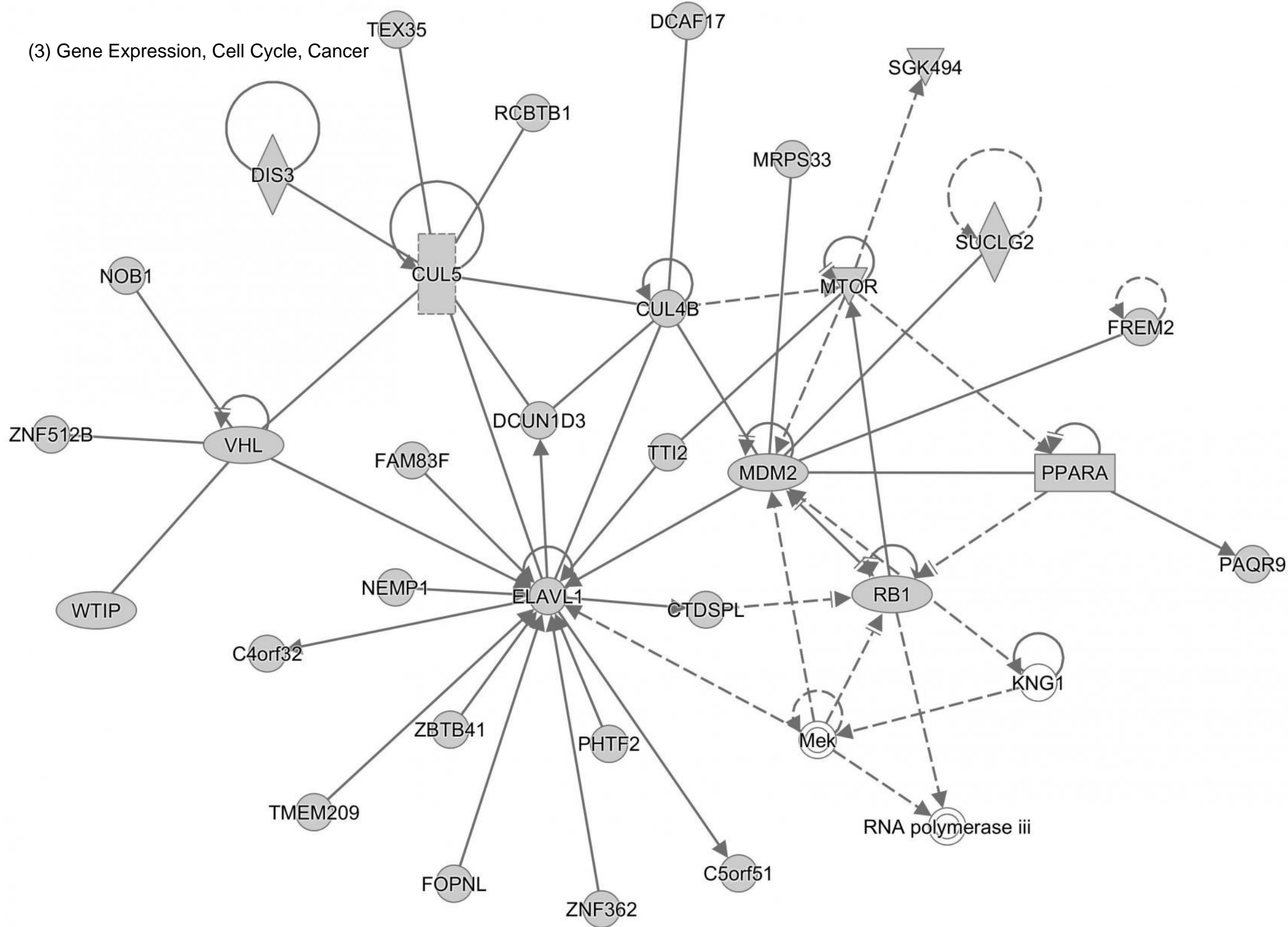

Supplement: Supplementary file 4 — Gene networks most relevant for lipid metabolism that were constructed by IPA using the miRNA’s target genes list. Grey shapes represent target genes and the white shapes are other genes of the network that are not target genes. (PDF 915 kb) [file 12864_2018_4514_MOESM4_ESM.pdf]

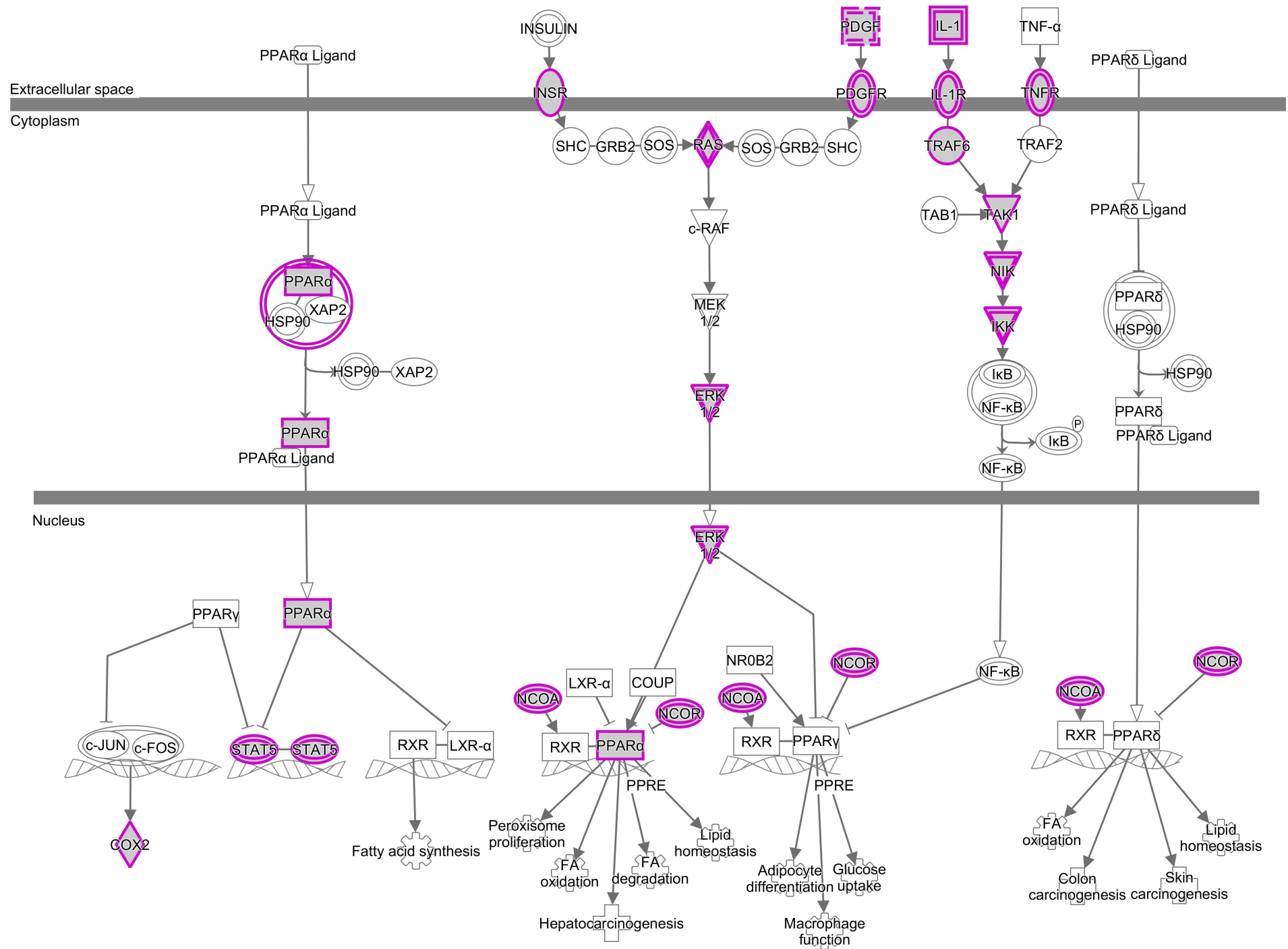

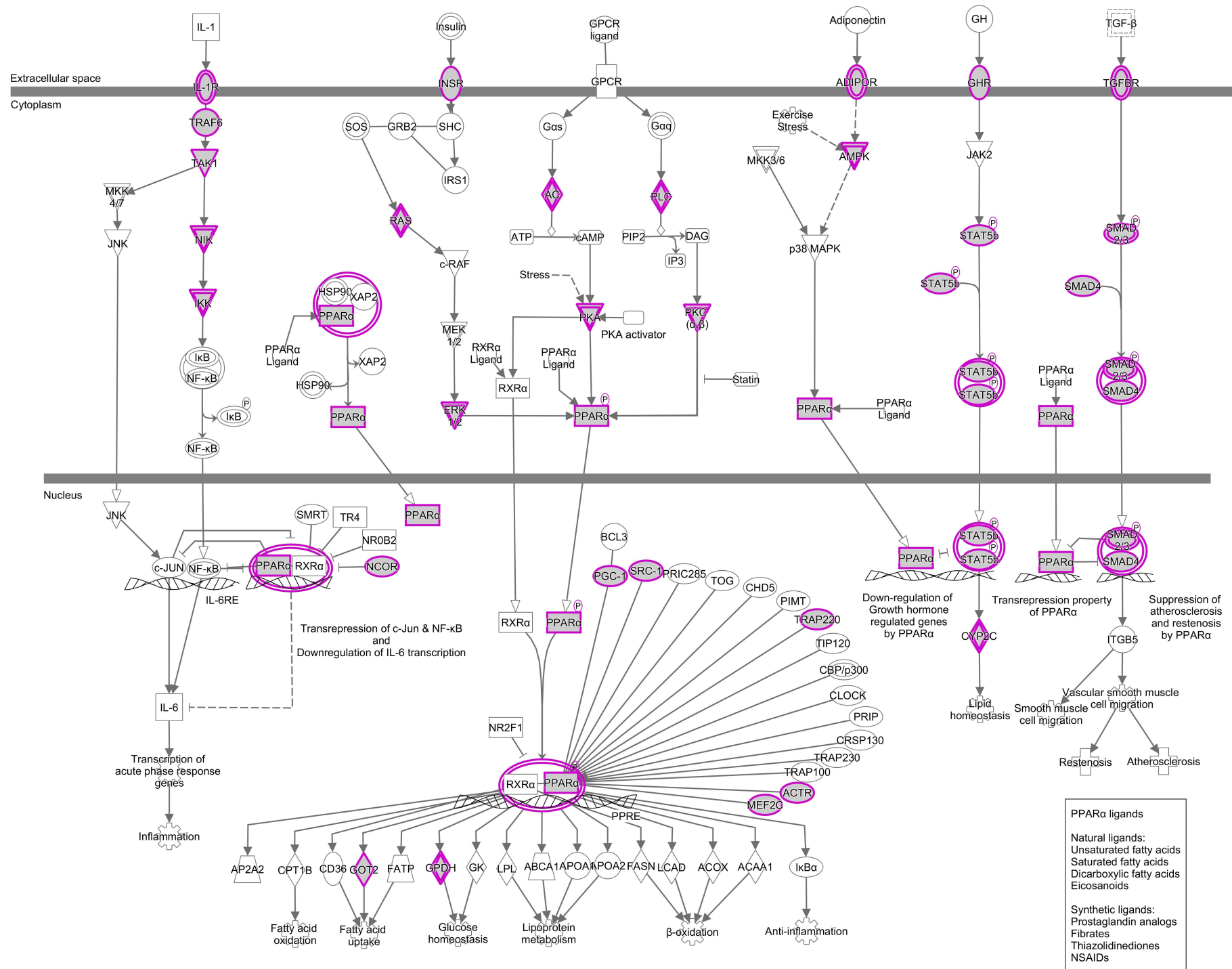

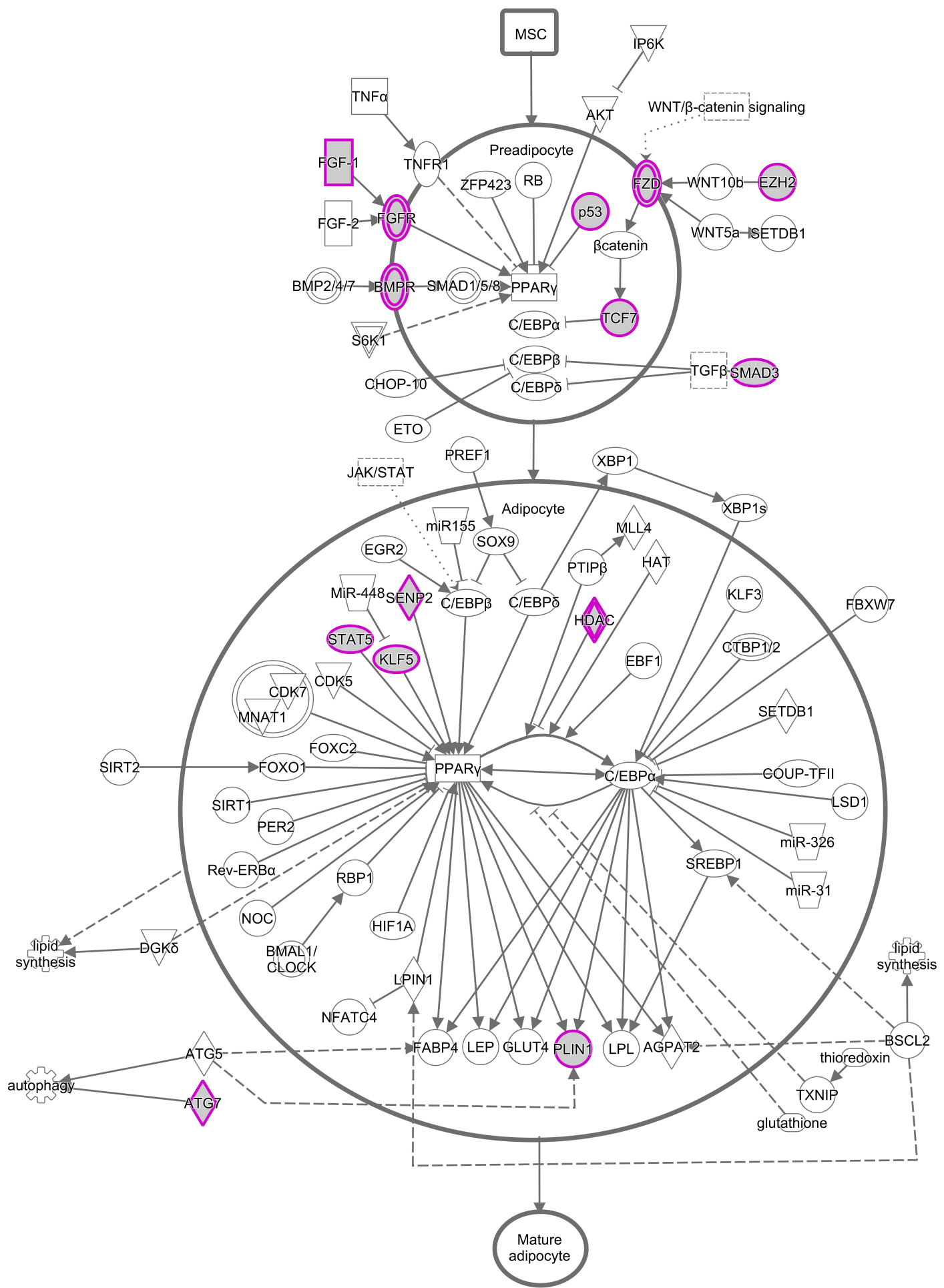

Supplement: Supplementary file 5 — The most relevant pathways enriched by IPA using the miRNA’s target genes list. The shapes highlighted in purple represent the miRNAs target genes and the white shapes represent the other genes of the pathway that are not target genes. (PDF 3964 kb) [file 12864_2018_4514_MOESM5_ESM.pdf]

**A****H-Module-trait relationships**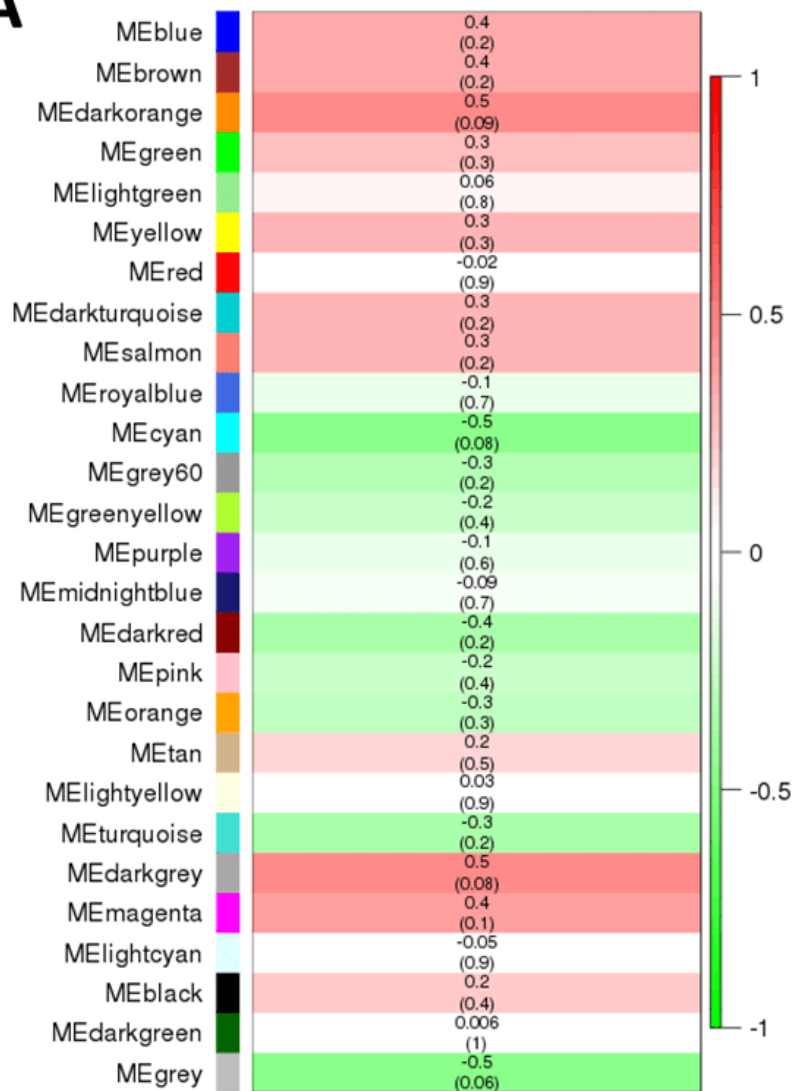**B****L-Module-trait relationships**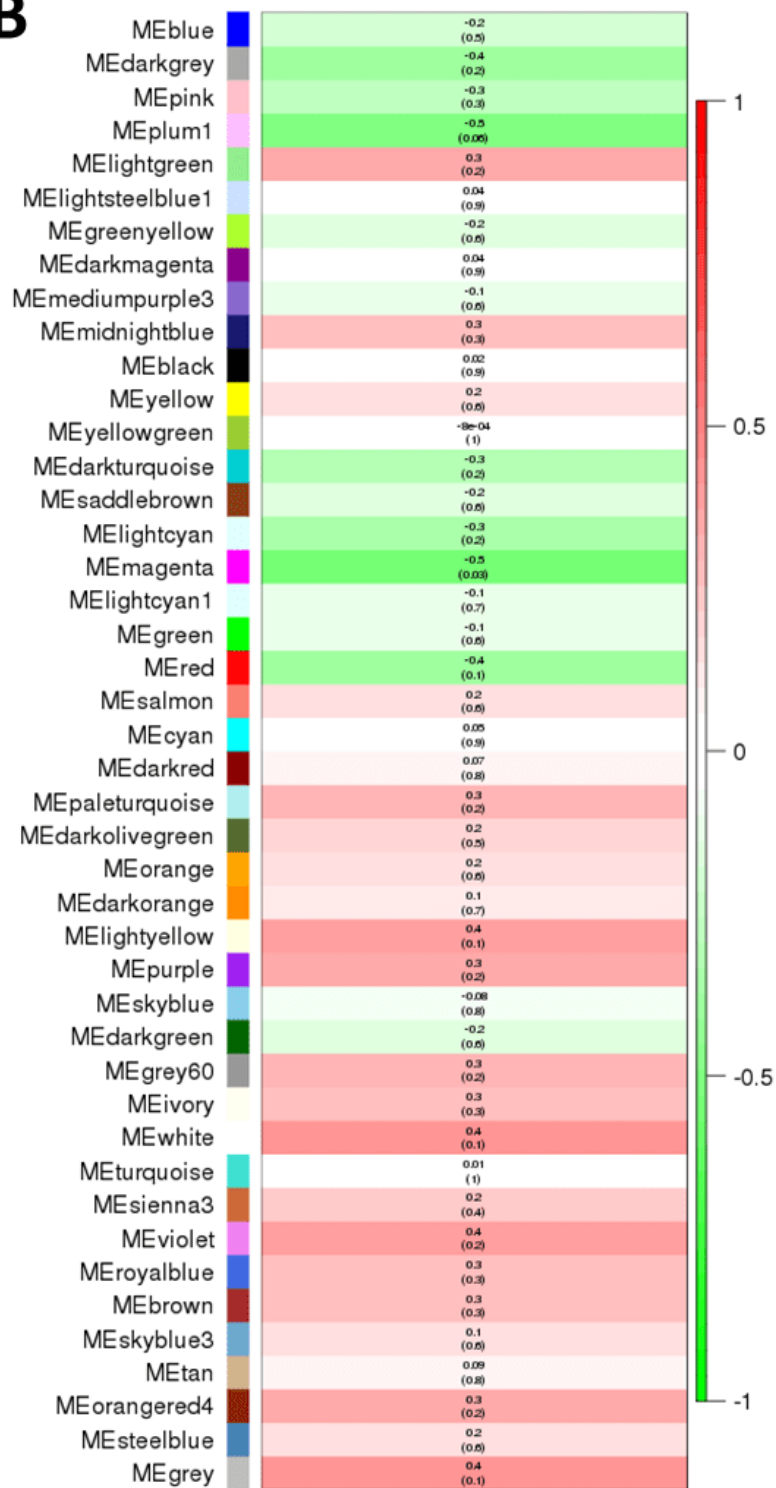

Supplement: Supplementary file 15 — Correlation between the mRNA modules and IMF. A: modules identified in the High IMF GEBV (H) group. B: modules identified in the Low IMF GEBV (L) group. Modules with intense red color have a higher correlation (close to + 1) and those with intense green color have a more negative correlation (close to − 1). (PDF 173 kb) [file 12864_2018_4514_MOESM15_ESM.pdf]

**A****H-Module-trait relationships**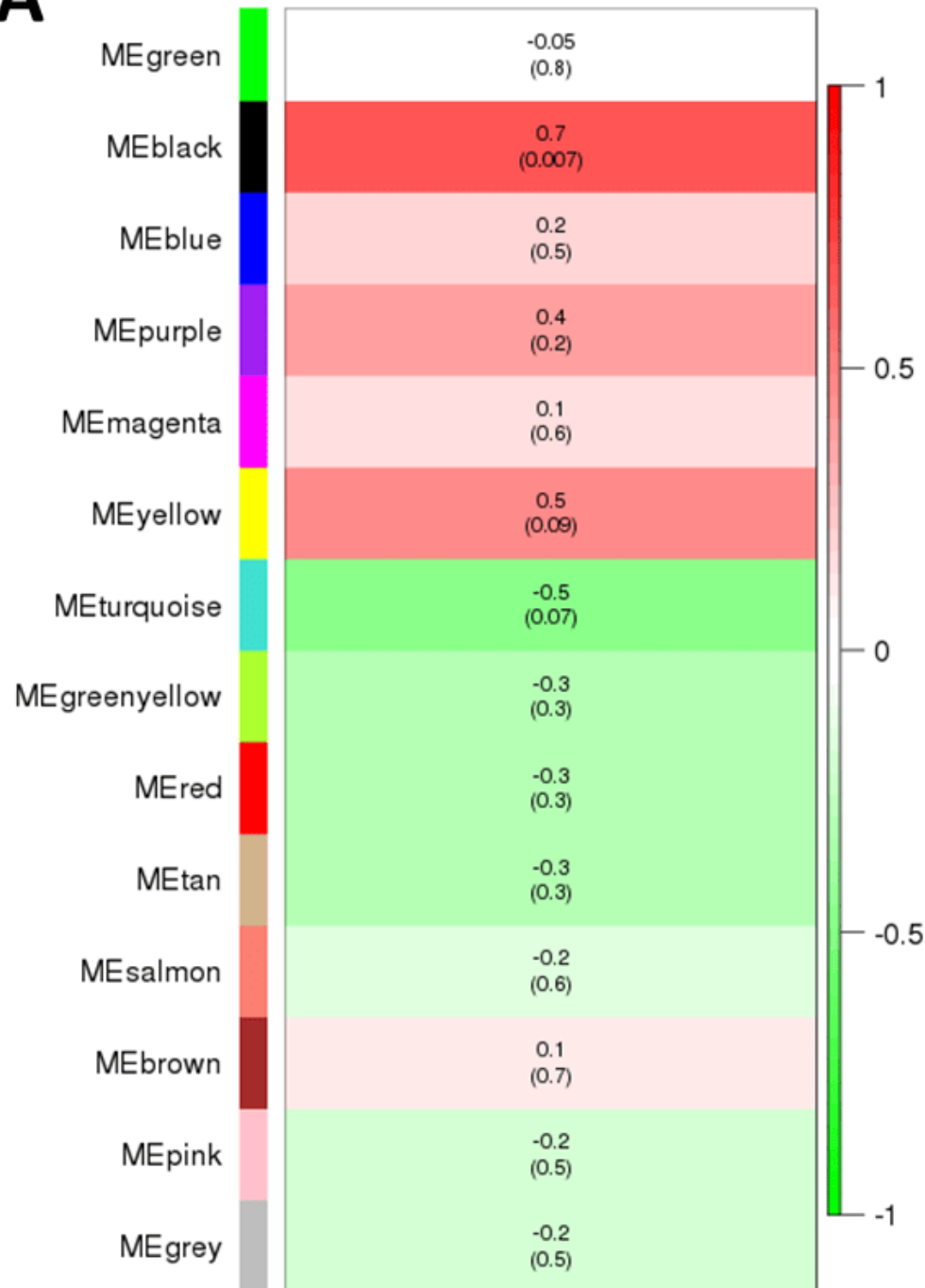**B****L-Module-trait relationships**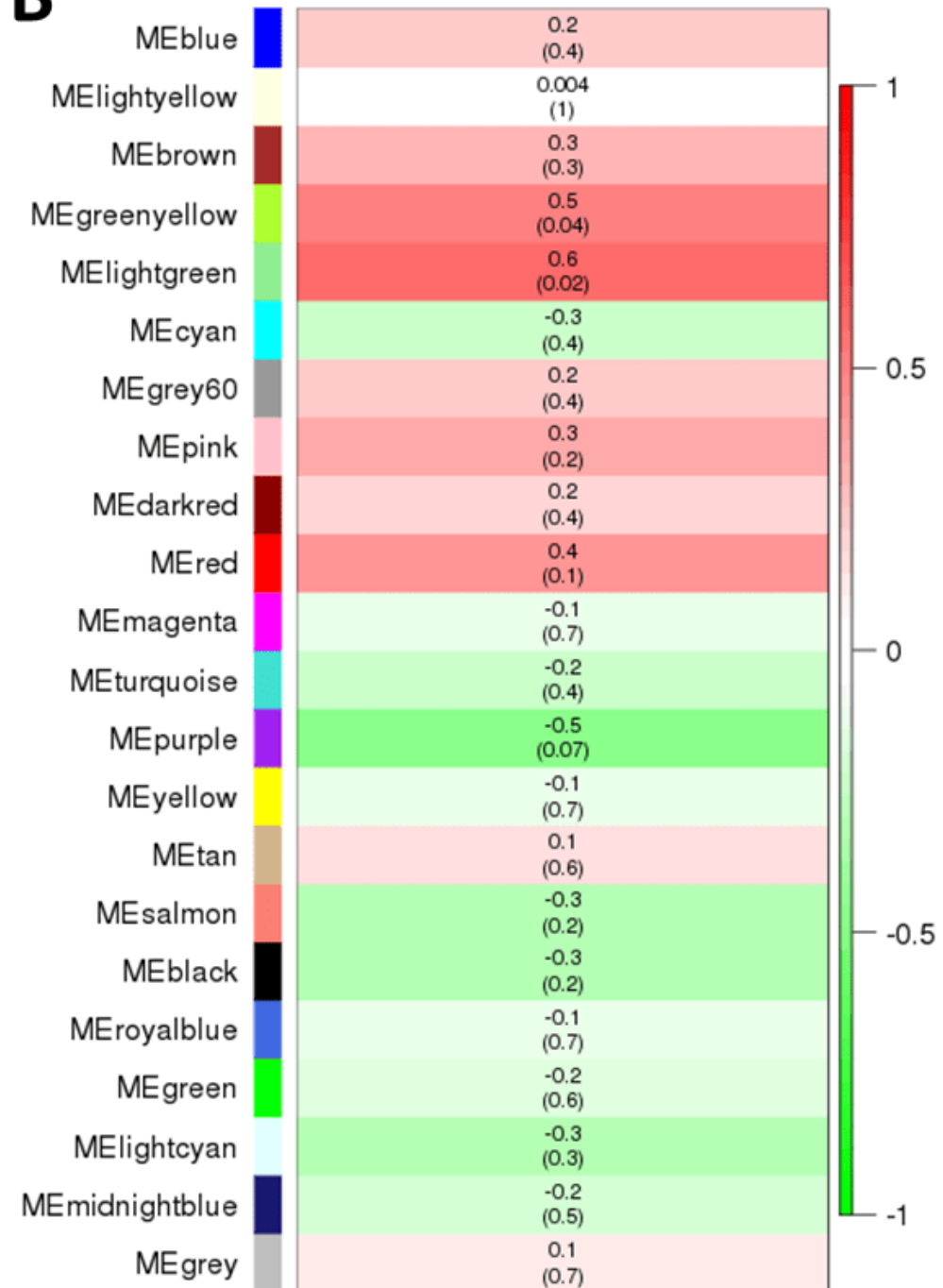

Supplement: Supplementary file 16 — Correlation between miRNA modules and intramuscular fat (IMF). A: modules identified in the High IMF GEBV (H) group. B: modules identified in the Low IMF GEBV (L) group. Modules with intense red color have a higher positive correlation (close to + 1) and those with an intense green color have a more negative correlation (close to − 1). (PDF 118 kb) [file 12864_2018_4514_MOESM16_ESM.pdf]

**A****Scale independence**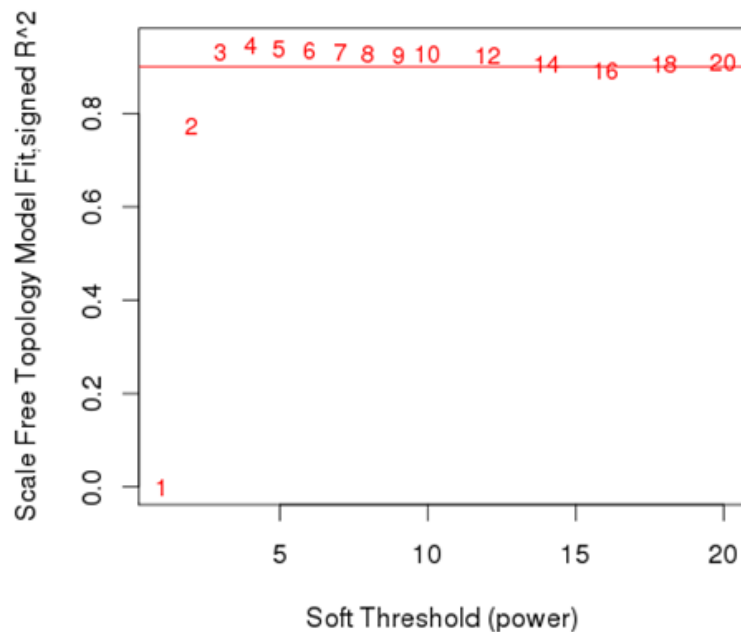**Mean connectivity**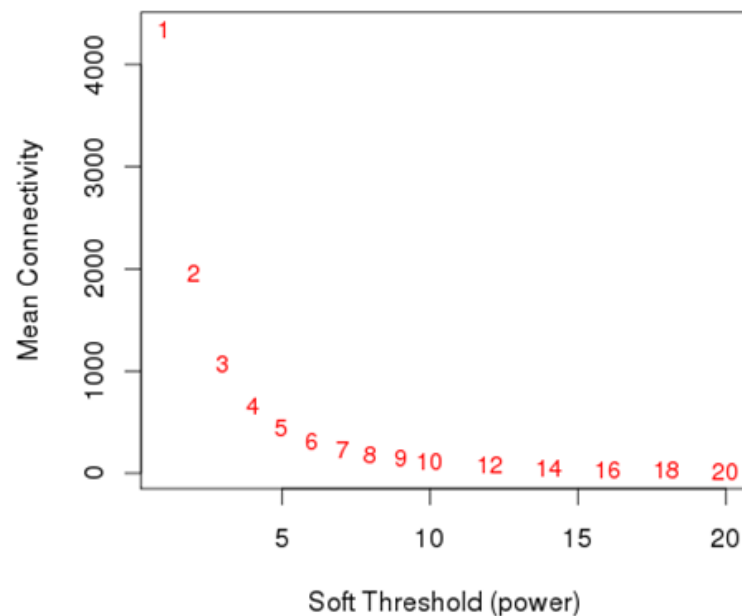**B****Scale independence**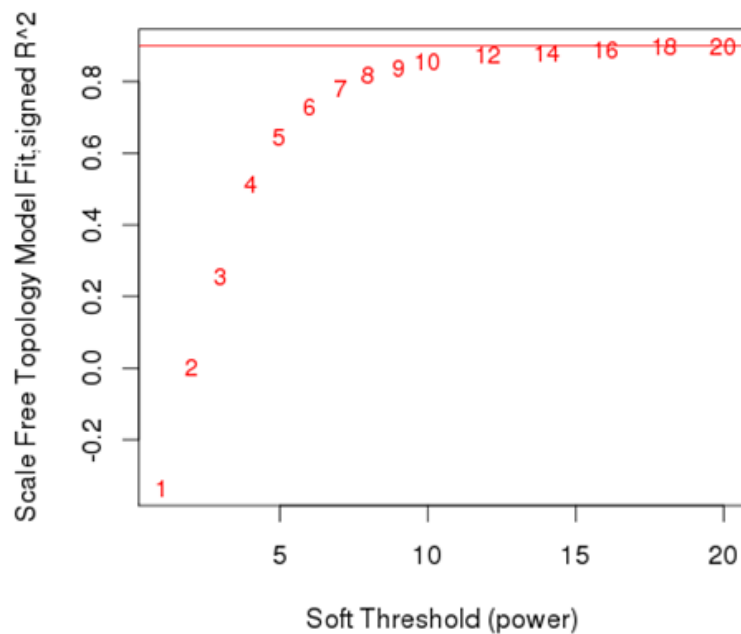**Mean connectivity**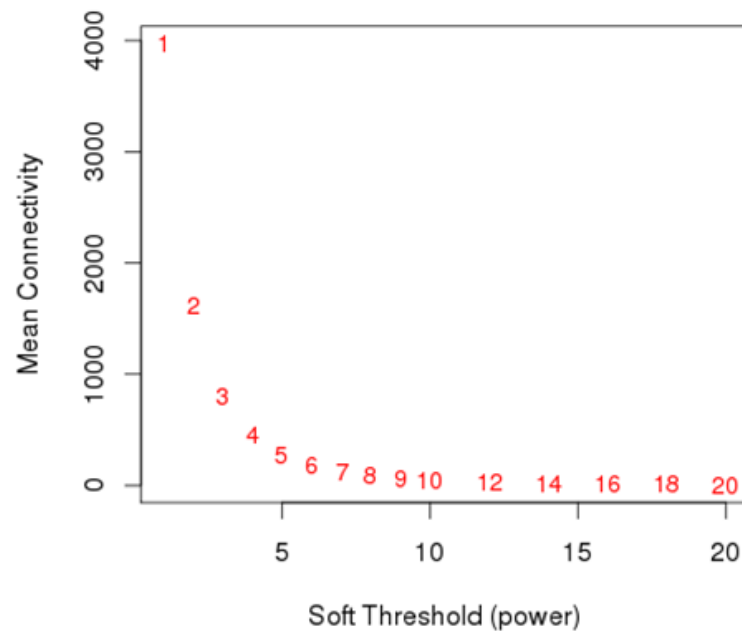

Supplement: Supplementary file 18 — Scale free topology model and mean connectivity of the mRNA networks based on the power value β. (A) H group. (B) L group. (PDF 92 kb) [file 12864_2018_4514_MOESM18_ESM.pdf]

**A**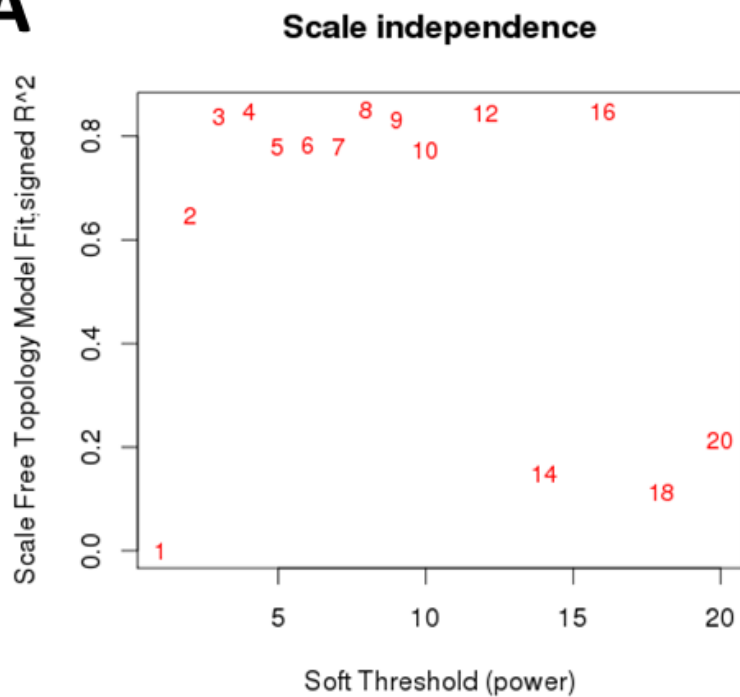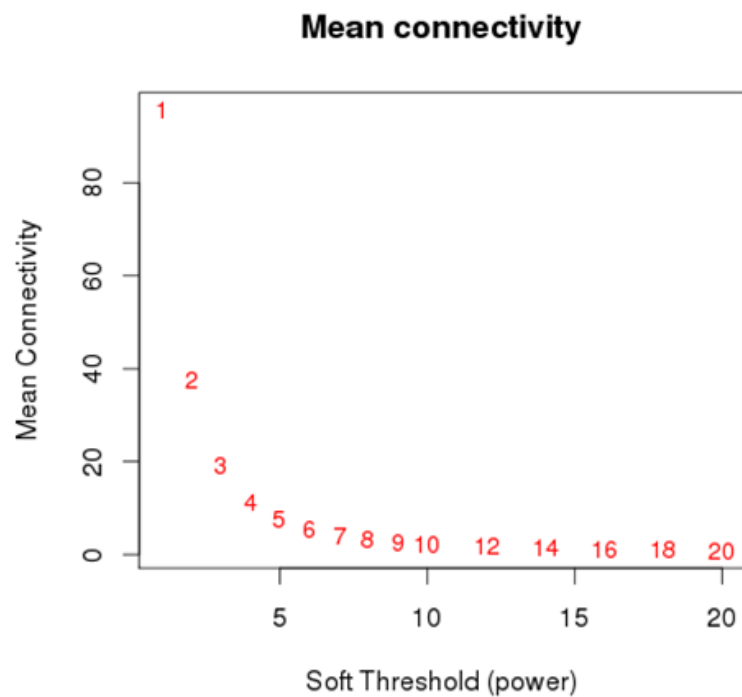**B**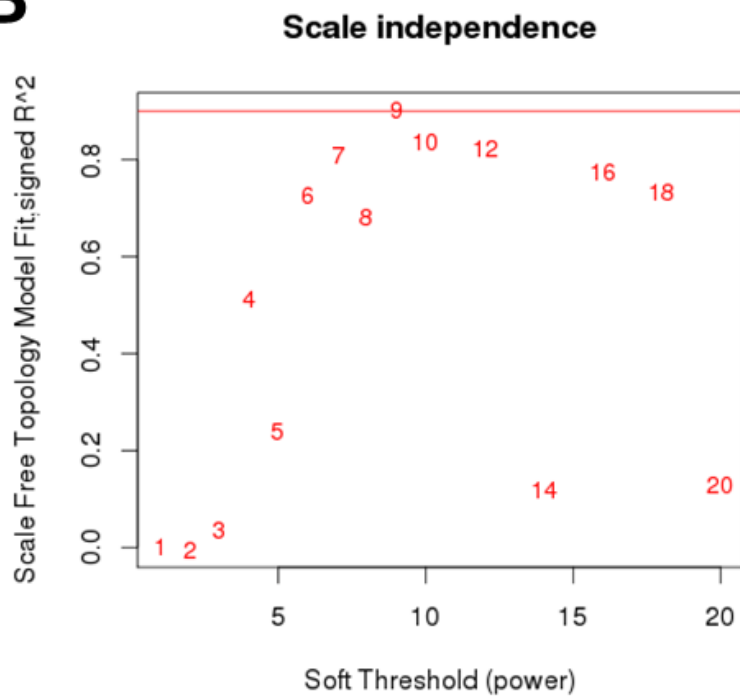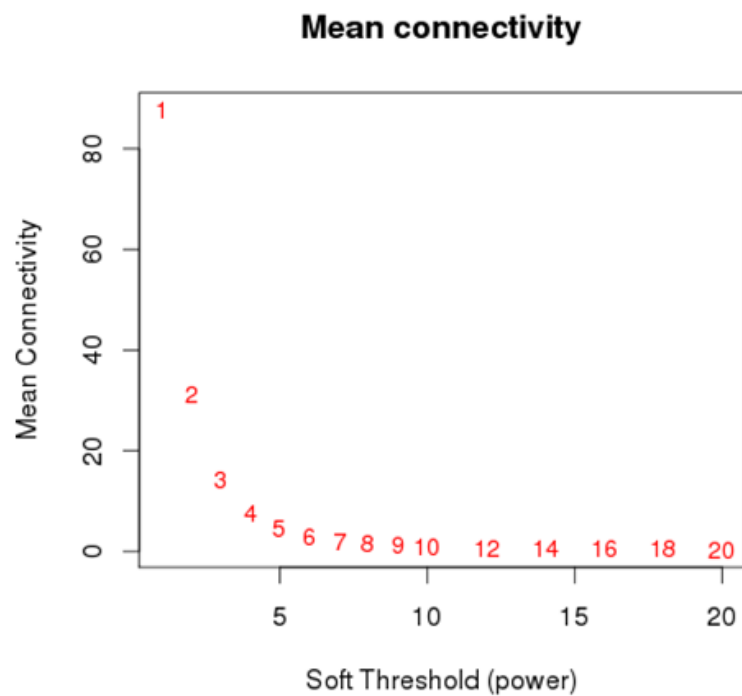

Supplement: Supplementary file 19 — Scale free topology model and mean connectivity of the miRNA networks based on the power value β. (A) H group. (B) L group. (PDF 85 kb) [file 12864_2018_4514_MOESM19_ESM.pdf]
